# Regulation of PVT‐CeA Circuit in Deoxynivalenol‐Induced Anorexia and Aversive‐Like Emotions

**DOI:** 10.1002/advs.202417068

**Published:** 2025-02-28

**Authors:** Liu‐Nan Yang, Mingmeng Tang, Andreas K. Nüssler, Liegang Liu, Wei Yang

**Affiliations:** ^1^ Department of Nutrition and Food Hygiene Hubei Key Laboratory of Food Nutrition and Safety Tongji Medical College Huazhong University of Science and Technology Hangkong Road 13 Wuhan 430030 China; ^2^ Department of Nutrition and Food Hygiene and MOE Key Lab of Environment and Health School of Public Health, Tongji Medical College Huazhong University of Science and Technology Hangkong Road 13 Wuhan 430030 China; ^3^ NHC Specialty Laboratory of Food Safety Risk Assessment and Standard Development Hangkong Road 13 Wuhan 430030 China; ^4^ Department of Traumatology BG Trauma Center University of Tübingen Schnarrenbergstr. 95 72076 Tübingen Germany

**Keywords:** anorexia, aversive‐like emotions, central amygdala (CeA), deoxynivalenol (DON), paraventricular thalamus (PVT)

## Abstract

Neuronal plasticity in the central amygdala (CeA) is essential for modulating feeding behaviors and emotional responses, potentially influencing reactions to Deoxynivalenol (DON). Acute oral administration of DON elicits a dose‐responsive reduction in food intake, accompanied by pronounced alterations in locomotor activity and feeding frequency. This study investigates circuitry adaptations that mediate DON's effects on feeding, by targeting of GABA neurons in the CeA. Following exposure to DON, an increase in connectivity between the paraventricular nucleus of the thalamus (PVT) and CeA^GABA^ neurons is observed, suggesting the involvement of this pathway in DON's adverse effects on feeding and emotional states. Chemogenetic and optogenetic manipulations of CeA^GABA^ neurons resulted in substantial alterations in mice's feeding and overall activity. These findings suggest that CeA^GABA^ neurons are involved in DON‐induced anorexia and aversive‐like emotional responses. Additionally, the administration of the SCN10A antagonist (A‐803467) effectively mitigated DON‐induced anorexia and aversive‐like emotions, highlighting the pivotal role of the PVT‐CeA circuit and CeA^GABA^ neurons in regulating the physiological and emotional impacts of DON. These findings have significant implications for public health and clinical interventions, offering potential therapeutic strategies to mitigate DON's adverse effects on human health.

## Graphical abstract

The study investigates Deoxynivalenol (DON) effects on feeding and emotions, focusing on the central amygdala (CeA). Acute DON exposure reduces food intake and alters behavior, linked to CeA^GABA^ neuron activity and PVT‐CeA connectivity. Manipulating CeA^GABA^ neurons and using SCN10A antagonist A‐803467 alleviate DON's effects, offering potential strategies for mitigating its impact.

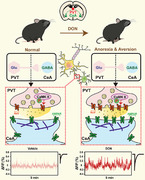

## 1 Introduction

The intricate interplay between appetite and emotion has been a longstanding focus of scientific inquiry. Food intake regulation is a multifaceted process, involving the coordinated interaction of peripheral signals and central regulatory circuits to acquire sufficient energy and nutrients for normal physiological functions.^[^ ^1^, ^2^ ^]^ Anorexia represents a pathological condition characterized by persistent food intake restriction, leading to extremely low body weight and often accompanied by an overwhelming preoccupation with weight and body shape.^[^ ^3^ ^]^ This condition can trigger severe health complications, encompassing malnutrition and organ dysfunction.^[^ ^4^, ^5^ ^]^ Remarkably, anorexia often co‐occurs with emotional and affective disturbances, prominently featuring aversive‐like emotions characterized by pronounced repulsion or rejection of specific objects, foods, or situations.^[^ ^6^, ^7^ ^]^

Previous outstanding studies have unveiled that specific mycotoxins and pharmaceutical agents not only disrupt the regulation of appetite, but also evoke extremely harmful emotional responses in individuals, including intense feelings of aversive and, at times, even hysteria.^[^ ^8^, ^9^ ^]^ Among various substances, Deoxynivalenol (DON), a mycotoxin produced by Fusarium species such as *F. graminearum* and *F. culmorum*, has been identified as a significant agent impacting appetite, neurological functions, and the immune system through complex mechanisms in different ages and genders models.^[^ ^10^, ^11^, ^12^, ^13^ ^]^ This may contribute to the development of anorexia and the emergence of negative mood states. For example, acute exposure to DON (1 or 2.5 mg kg^−1^ body weight) has been shown to induce anorexia in mice within two hours,^[^ ^14^, ^15^ ^]^ stimulating the brain's anorexia center.^[^ ^16^ ^]^ However, the detailed mechanisms and comprehensive understanding of DON‐induced anorexia and adverse effects remain limited.

Recently, many studies have focused on the hypothalamus as a central hub in anorexia, but other regions within the central nervous system have also emerged as critical factors in the regulation of appetite and emotions.^[^ ^17^, ^18^ ^]^ Notably, the central amygdala (CeA), acting as a specific region of brain, is either directly or indirectly implicated in hypothalamic regulation and exerts a substantial influence over emotional and food intake behaviors.^[^ ^19^, ^20^ ^]^ The CeA activity not only regulates food preferences, but is also intricately intertwined with emotional regulation, encompassing emotions akin to aversive.^[^ ^21^ ^]^ Besides, alterations in CeA activity levels have been shown to modulate emotional responses to diverse stimuli, sometimes culminating in anxiety and repulsion, particularly when over‐activated.^[^ ^22^ ^]^ Consequently, the CeA may emerge as a crucial nexus, interlinking key nodes between anorexia and aversive‐like emotions in a neurobiological context. Meanwhile, another region of interest is the paraventricular thalamus area (PVT), which also plays a pivotal role in the regulation of both appetite and emotions.^[^ ^23^, ^24^ ^]^ PVT regulates the initiation and cessation of food intake behaviors through the detection of food rewards, hunger signals, and external stimuli, thus exerting a substantial influence on affective states.^[^ ^25^, ^26^, ^27^, ^28^ ^]^ The interaction between PVT and CeA may significantly contribute to the regulation of anorexia and aversive‐like emotions. However, related research regarding mechanism of interaction between PVT and CeA in DON‐induced anorexia is also limited.

This study delves into the neuromodulatory mechanisms of the PVT‐CeA circuit in DON‐induced anorexia, and examining its influence on emotion regulation and aversive‐like behaviors. To achieve this objective, several advanced techniques such as viral tracing, in vivo/ex vivo electrophysiology, and neurogenetics were employed in this study to initially discover the role of GABAergic neurons within this circuit. Simultaneously, our aim also is to unravel the PVT‐CeA circuit's regulatory functions in the context of DON‐induced anorexia, providing potentially mechanism and guiding strategies to mitigate the toxic effects. Collectively, this research underscores the ongoing imperative to control dietary DON levels, aligning with broader public health objectives.

## 2 Results

### 2.1 Acute DON Treatment Evoke Food Intake Reduction and Aversive‐Like Emotions

**Figure 1:**
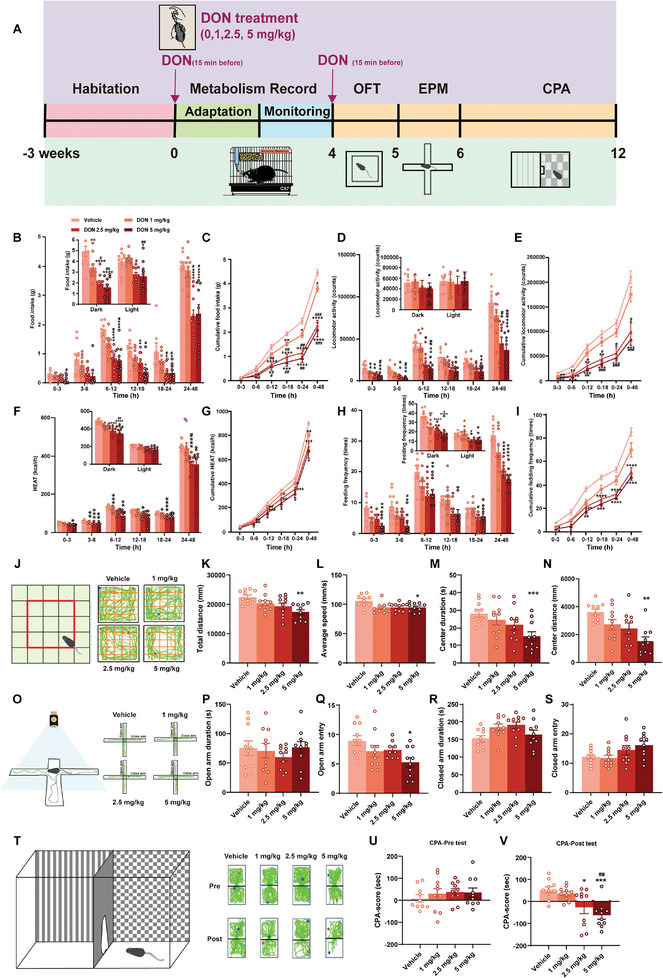
DON‐Induced Physiological and Behavioral Changes in Mice A) Schematic of experimental design. (B‐C) Food Intake: Tracked at intervals B) and cumulatively C) over 48 h in mice treated with vehicle or DON (1, 2.5, and 5 mg kg^−1^) orally in 8‐week‐old female C57BL/6J mice. Inset details nocturnal/diurnal food intake. D‐E) Locomotor Activity: Recorded over 48 h, with nocturnal/diurnal activity patterns in inset. F‐G) HEAT: Assessed at intervals (F) and cumulatively (G) over 48 h. Inset shows nocturnal/diurnal HEAT. H‐I) Feeding Frequency: Monitored over 48 h; nocturnal/diurnal frequency shown in inset. J‐N) OPT test: Includes representative heatmaps and schematics (J), total distance (K), average speed (L), center duration (M), and center distance (N). O‐S) EPM test: Includes representative heatmaps and schematics (O), open arm duration (P), open arm entries (Q), close arm duration (R), and close arm entries (S). T‐V) CPA test: Includes representative heatmaps and schematics (T), pre‐test (U), and post‐test (V) data. Note: Behavioral tests initiated 15 min post‐gavage; trajectory endpoints marked in red (start) and blue (end). n = 10–11 mice per group. Statistical Methods: Employed one‐way ANOVA (K‐S, U‐V) and two‐way ANOVA (B‐I) for data analysis.

In this study, 8‐week‐old female C57/BL6J mice, post‐acclimatization, underwent assessment in the CLAMS system (**Figure** **1**A). Within 48 h, significant dose‐dependent reductions in food intake (Figure 1B) and cumulative food intake (Figure 1C) were observed with oral DON treatment (1, 2.5, and 5 mg kg^−1^), administered to the mice via gavage 15 min before each experiment. Notable differences were particularly detected in the first 0–3 h between 1 mg kg^−1^ and 5 mg kg^−1^ DON (*p* < 0.05), and between 6–12 h for the vehicle compared to 2.5 mg kg^−1^ (*p* < 0.0001) and 5 mg kg^−1^ DON (*p* < 0.001) for up to 48 h. Although no significant difference was noted between the vehicle and 1 mg kg^−1^ DON (*p* = 0.8075), a notable reduction in food consumption occurred 6 hours after all three doses. Parallel to food consumption, water intake in DON‐treated and vehicle mice was quantified (Figure S1A‐B, Supporting Information), with higher DON doses significantly reducing water intake over 48 h: vehicle compared to 2.5 mg kg^−1^ DON (*p* < 0.001) and 5 mg kg^−1^ DON (*p* < 0.001). Cumulative consumption monitoring confirmed DON's anorectic effect, while locomotor activity and HEAT (kcal h^−1^) changes (Figure 1D‐G) indicated typical anorexia characteristics due to pathological condition. Activity significantly reduced within 0–3 h, especially with 2.5 mg kg^−1^ (*p* < 0.01) and 5 mg kg^−1^ DON (*p* < 0.01), persisted after 24 h (Figure 1D‐E). HEAT differed significantly across all DON doses over 48 hours, with notable differences at 3–6 h for the vehicle and 1 mg kg^−1^ DON (*p* < 0.01) and 6–12 h for the vehicle and 2.5 mg kg^−1^ DON (*p* = 0.0031). Whole‐body oxygen consumption (VO_2_), Whole‐body carbon dioxide consumption (VCO_2_), respiratory entropy (RER, ratio of VCO_2_ and VO_2_), and feeding frequency also demonstrated dose‐dependent declines (Figure S1C‐D,E‐F,G‐H; Figure 1H‐I, Supporting Information) at various intervals, such as 0–3 h and 12–18 h.

Behavioral assessments using OFT (Figure 1J‐N) revealed significant movement reduction in the 5 mg kg^−1^ DON group compared to the vehicle. EPM testing (Figure 1O‐Q) showed no significant differences in anxiety‐like behaviors, although the number of open arm entries significantly decreased with 5 mg kg^−1^ DON (Figure 1Q), suggesting inhibitory effects at higher doses. CPA testing (Figure 1T‐U) indicated a significant aversion in higher dose groups (2.5 and 5 mg kg^−1^), highlighting a pronounced aversive response to DON treatment. These findings demonstrate that DON significantly impacts food intake, locomotion, and feeding behavior, resulting in anorexia. The observed changes in locomotion preclude a definitive conclusion regarding DON's capacity to induce acute anxiety. Instead, the CPA data suggests that DON effectively induces a conditioned place aversion.

### 2.2 CeA^GABA^ Neurons Mediate the Negative Emotional Effects Induced by DON

**Figure 2:**
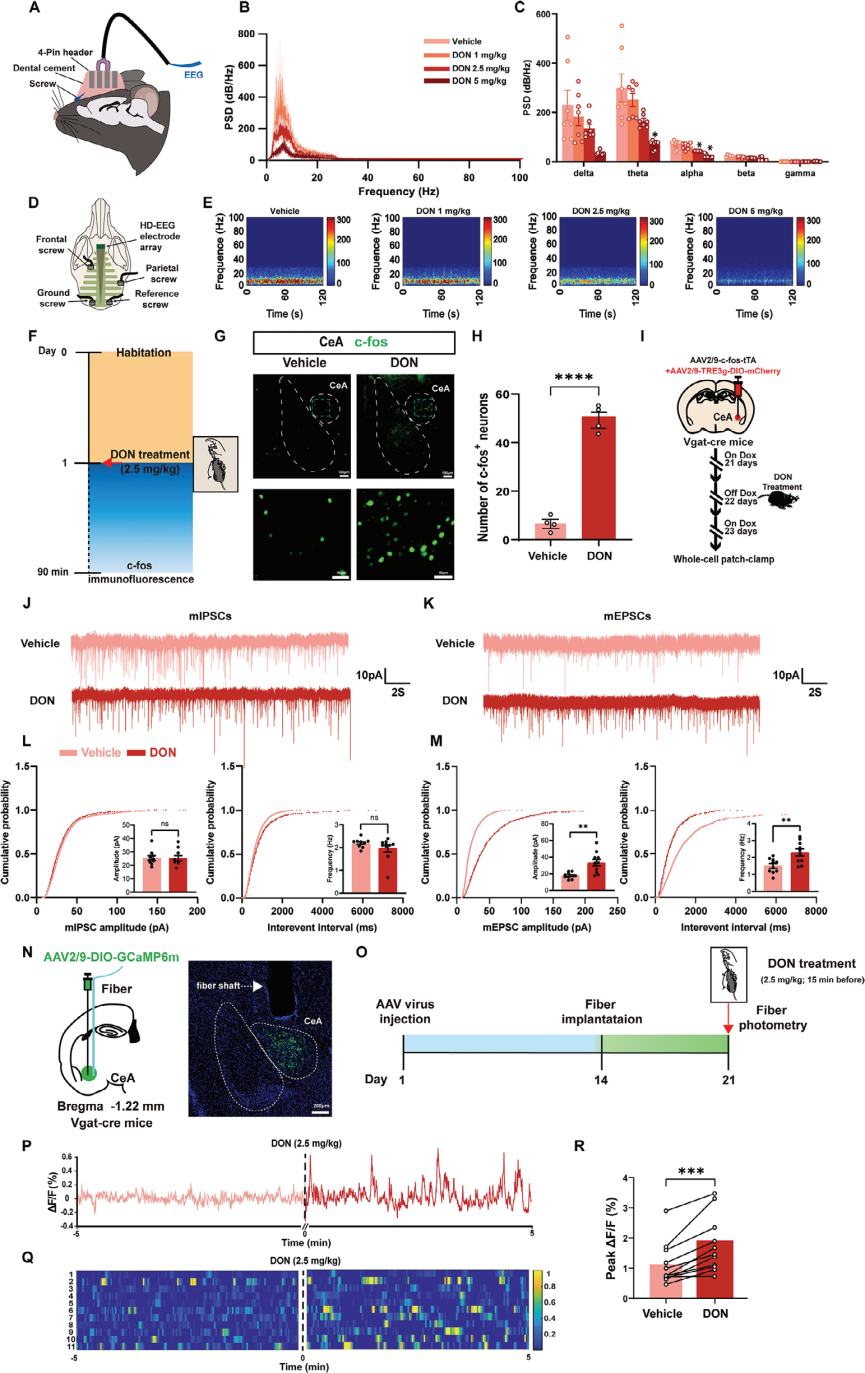
DON‐Induced Neurophysiological Changes in Mice A) EEG Schematic. B) PSD Analysis: Power spectral density in Vehicle and DON‐treated groups (1, 2.5, and 5 mg kg^−1^). C) PSD Quantification: Notable increases in theta and alpha band energy in higher DON doses. Statistical analysis using two‐way ANOVA. D) EEG electrode placement diagram. E) EEG spectrograms: representative power spectrograms across all groups. Time 0 marks saline and DON administration. F) Schematic of c‐fos procedure. G) c‐fos Expression: Visualized in CeA of Vehicle and DON 2.5 mg kg^−1^ groups. Scale: 100 µm. H) c‐fos Quantification: Statistical analysis of c‐fos‐positive cells showing significant increase in DON group (Welch‐corrected two‐tailed unpaired t‐test, P < 0.0001). n = 4 mice per group. I) Schematic Diagram of Tet‐off Viral System Combined with Whole‐Cell Patch‐Clamp Electrophysiology J) Representative mIPSC traces. K) Representative mEPSC traces. L) Cumulative distribution of mIPSC amplitude (left) and interevent intervals (right). Inset, mean Amplitude (left) and Frequency (right) of mIPSCs in mice (n  =  10 cells from four mice; two‐tailed unpaired t‐test; frequency, *p*  =  0.2050; amplitude, *p*  =  0.9068). M) Cumulative distribution of mEPSC amplitude (left) and interevent intervals (right). Inset, mean Amplitude (left) and Frequency (right) of mEPSCs in mice (n  = 10 cells from four mice; two‐tailed unpaired t‐test; frequency, *p*  =  0.0045; amplitude, *p*  =  0.0017). N) Schematic (left), and representative (right) CeA injection sites in Vehicle and DON 2.5 mg kg^−1^ group for fiber photometry recording. Scale: 50 µm. O) Fiber photometry procedure. P) Traces of calcium fluorescence dynamics following DON treatment (pink trace for pre and red trace for post) in CeA. Q) Heatmap visualized the changes of calcium fluorescence signals in CeA (n = 11 mice per group). R) Diagram showing the peak ΔF/F0 for pre and post treatment of DON treatment (n = 11 mice per group, two‐tailed unpaired t‐test, *p* = 0.0004).

To elucidate the effects of DON on brain neural activity, cortical electroencephalograms (EEGs) were conducted on the frontal cortex of mice before and after administration of DON (**Figure** **2**A‐E). Post‐DON exposure EEG analysis revealed a dose‐dependent attenuation of theta and alpha wave activities. Notably, at higher doses (5 mg kg^−1^), significant reductions in theta waves were observed, suggesting potential impairments in cognitive functions, memory, and emotional processing. Similarly, decreases in alpha wave activities at doses of 2.5 and 5 mg kg^−1^ (*p* = 0.0423 and *p* = 0.0128, respectively, compared with the Vehicle) indicated a reduction in overall brain activity and processing capabilities. These effects underscore the profound adverse impact of DON on brain function starting from 2.5 mg kg^−1^, with exacerbated effects at higher doses (Figure 2B‐C).

Based on the initial observations of DON's effects on cortical regions, our investigation further sought to delineate its functional impacts on distinct brain areas. The identification of the CeA, pivotal in modulating emotional and feeding responses,^[^ ^19^, ^29^, ^30^, ^31^ ^]^ implies that DON's reach likely extends to critical neural pathways that govern emotional and behavioral regulation. We examined the effects of DON on several key brain regions involved in regulating emotional responses and food intake.^[^ ^31^, ^32^, ^33^, ^34^ ^]^ A fluorescent brain‐wide activity map induced by DON revealed that the CeA exhibited particularly significant neuronal activity changes among all brain regions (Figure S2D‐H; Figure S3, Supporting Information). Although other brain regions such as the Anterior Cingulate Cortex (ACC), Nucleus Accumbens (NAcc), Bed Nucleus of the Stria Terminalis (BNST), and Ventral Tegmental Area (VTA) did not show significant activation following DON treatment (Figure S2D‐H, Supporting Information), while the Ventromedial Hypothalamic Nucleus (VMH) showed neuronal activation (Figure S2F, Supporting Information), additional experiments revealed that VMH activation did not induce anxiety‐like or aversive behaviors^[^ ^35^ ^]^ (Figure S4, Supporting Information). Based on these findings, we directed our investigation to the CeA. The CeA is uniquely involved in regulating both feeding behavior and emotional responses, particularly in the modulation of aversive and anxiety‐like emotions,^[^ ^29^, ^31^ ^]^ making it a more suitable target for studying the effects of 2.5 mg kg^−1^ DON on emotional and behavioral regulation.

A significant increase in c‐fos positive cells in the CeA compared to the vehicle group was observed (Figure 2G‐H). Subsequent in vivo electrophysiological recordings indicated a significant increase in delta wave basal discharge rates and a reduction in theta waves in the CeA following DON treatment (Figure S2A‐C, Supporting Information). This alteration may stem from heightened GABAergic neuron excitability. To investigate the characteristics of the neuronal ensemble activated by DON in the CeA^GABA^, we employed the Tet‐off viral system in combination with whole‐cell patch‐clamp electrophysiology (Figure 2I). A mixture of AAV2/9‐c‐fos‐tTA and AAV2/9‐TRE3g‐DIO‐mCherry was injected into the CeA of Vgat‐cre mice, followed by 2.5 mg kg^−1^ DON stimulation to selectively label activated neurons. Whole‐cell patch‐clamp techniques were employed to record minimal inhibitory postsynaptic currents (mIPSCs) and miniature excitatory postsynaptic currents (mEPSCs) in the CeA (Figure 2J‐K). Results demonstrated that DON notably increased the frequency and amplitude of mEPSCs in CeA pyramidal neurons (Figure 2K,M), while mIPSCs remained unaffected (Figure 2J,L). It indicated that DON treatment leads to augmented excitatory synaptic transmission in the CeA, primarily via neurotransmitters like glutamate, while transmission from inhibitory neurotransmitters, such as GABA, remains unaffected. Next, in vivo fiber photometry was employed to monitor intracellular Ca^2+^ transients, indicative of neuronal activity in GABA neurons. This involved the stereotaxic injection of AAV2/9‐DIO‐GCaMP6m into the CeA of Vgat‐cre mice (Figure 2N left), leading to the expression of GCaMP6m in CeA^GABA^ neurons (Figure 2N right,). Two weeks following the optical fiber cannula implantation at the same site (Figure 2O), significant increases in GCaMP6m fluorescence were detected in CeA^GABA^ neurons upon DON exposure (Figure 2P‐R), indicating enhanced activity of these typically inhibitory GABAergic neurons in the DON condition.

### 2.3 Chemogenetic Modulation of CeA^GABA^ Neurons in Regulating Anorexia and Aversive‐Like Emotions Induced by DON

**Figure 3:**
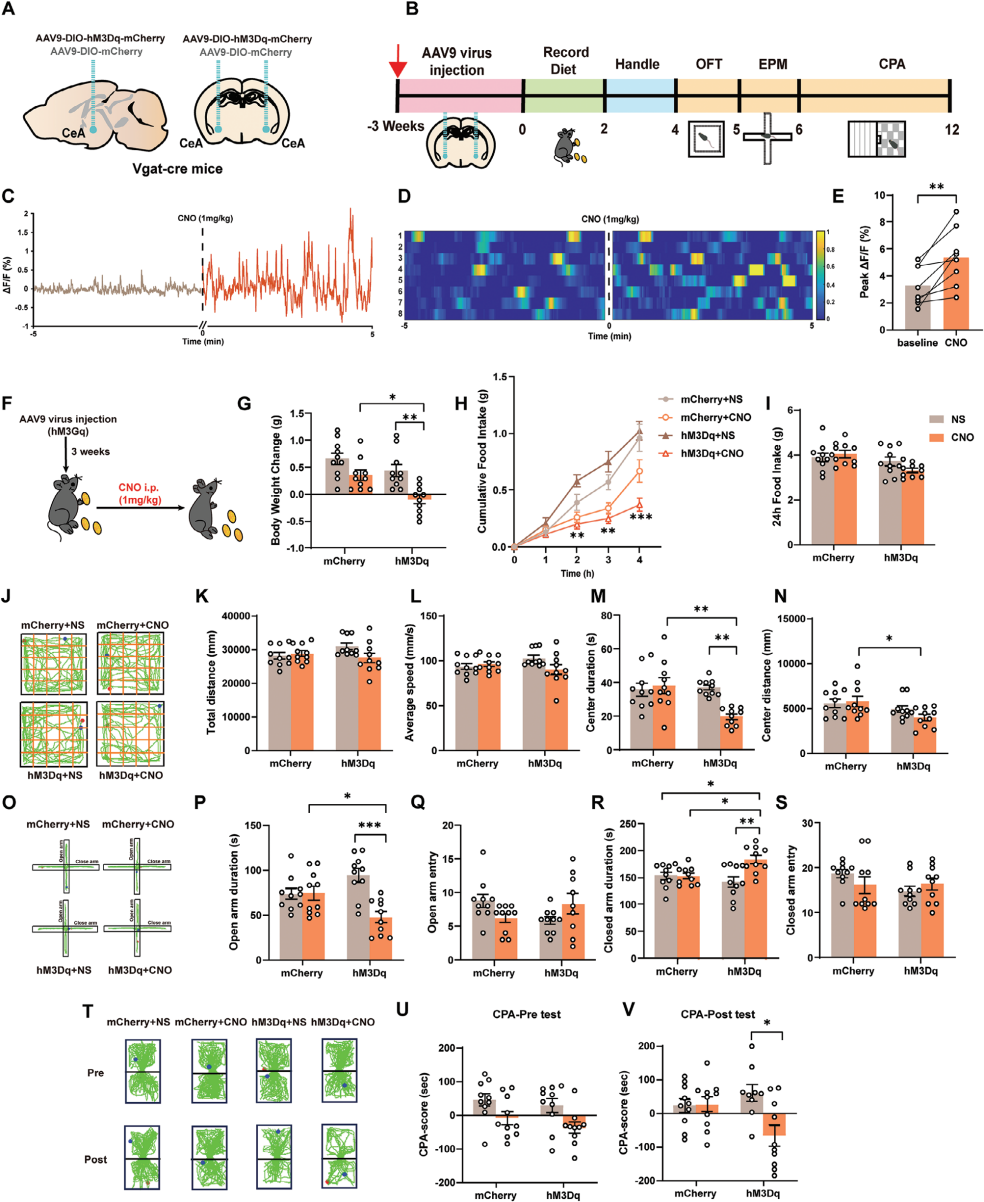
Chemogenetic Activation of CeA^GABA^ Neurons Triggers Anorexia and Aversive‐like Emotions A) Virus (AAV9‐hM3Dq‐mCherry) injection in Vgat‐cre mice's CeA nucleus. B) Experimental design and timeline. C) Traces of calcium fluorescence dynamics following CNO injection (brown trace for pre and red trace for post) in CeA. D) Heatmap visualized the changes of calcium fluorescence signals in CeA (n = 8 mice per group). E) Diagram showing the peak ΔF/F0 for pre and post CNO injection (n = 8 mice per group). F) Food Intake post‐CNO administration schematic. G) Body Weight Changes in 24 h. H) Body Weight Changes in 4 h. I) Body Weight Changes in 24 h. J) OPT Motion Trajectory. K) OPT Total Distance. L) OPT Average Speed. M) OPT Center Duration. N) OPT Center Distance. O) EPM Motion Trajectory. P) EPM Open Arm Duration. Q) EPM Open Arm Entry. R) EPM Close Arm Duration. S) EPM Close Arm Entry. T) CPA Motion Trajectory. U) CPA Pre‐Test. V) CPA Post‐Test. N = 10 mice per group G‐V). Statistical Methods: Employed one‐way ANOVA (G, I, K‐N, P‐S, U‐V), two‐way ANOVA (H), and Welch‐corrected two‐tailed unpaired t‐test (E) for data analysis.

In exploring the role of CeA^GABA^ neurons in DON‐induced behavioral changes, we employed chemogenetics for both activation and inhibition studies in Vgat‐cre mice. Activation involved bilateral infection with AAV9‐DIO‐hM3Dq‐mCherry in the CeA (**Figure** **3**A; Figure S5, Supporting Information), and Clozapine‐N‐Oxide (CNO) induced anorexia and aversive‐like behaviors (Figure 3B). Calcium imaging confirmed increased basal firing rates of GABA neurons after CNO administration, validating the activation approach (Figure 3C‐E, Figure S2I‐J, Supporting Information). This led to significant reductions in body weight and food intake (Figure 3F‐I), particularly notable from the second hour onwards compared to the mCherry+NS group (second hour: *p* = 0.0017, third hour: *p* = 0.0031, fourth hour: *p* = 0.0005; Figure 3H). In behavioral assessments (Figure 3J‐V), hM3Dq mice administered with CNO, compared with mCherry and NS groups, exhibited no significant change in total movement distance (Figure 3K) or average speed (Figure 3L) in the OFT. However, a notable reduction in both exploration time (Figure 3M) and distance traveled in the center area (Figure 3N) was observed (center duration: mCherry+CNO versus hM3Dq+CNO: *p* = 0.0013, hM3Dq+NS versus hM3Dq+CNO: *p* = 0.0027; center distance: mCherry+CNO versus hM3Dq+CNO: *p* = 0.0408). Additionally, CNO‐mediated activation of CeA^GABA^ neurons led to decreased time spent in open arms (Figure 3P) and increased duration in closed arms (Figure 3R) of the EPM. There were no significant differences in the number of entries into the open or closed arms (Figure 3Q,S). Furthermore, in the CPA test, hM3Dq mice treated with CNO displayed strong aversive emotions (Figure 3T‐V), demonstrating a marked reluctance to remain in the starting room compared to the saline‐injected group (hM3Dq+NS versus hM3Dq+CNO: *p* = 0.0135, Figure 3V).

**Figure 4:**
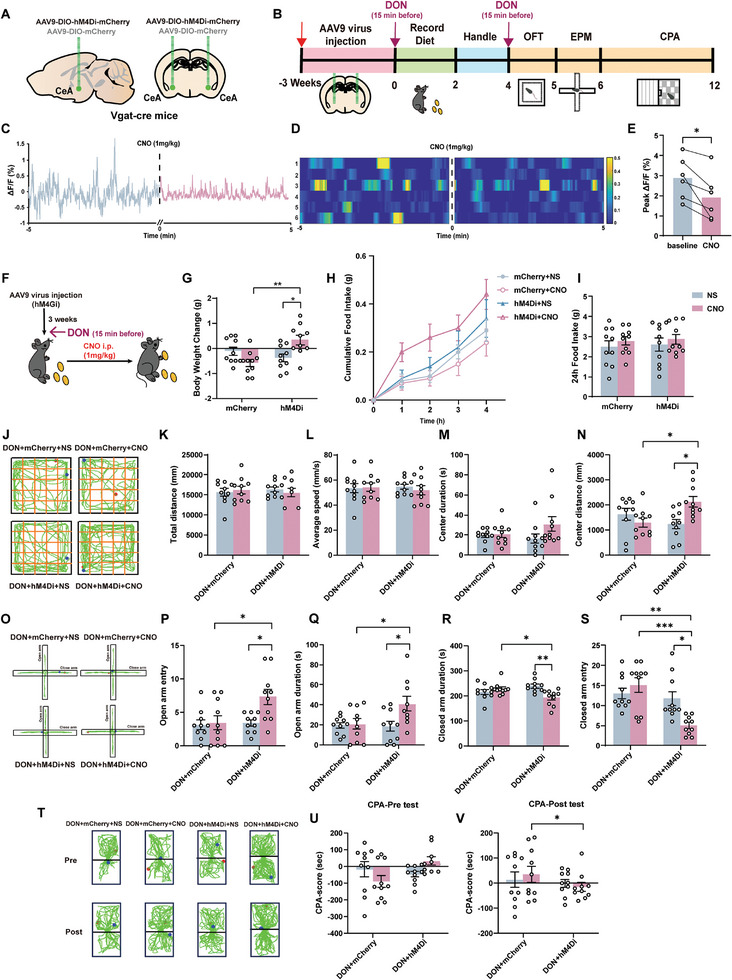
Chemogenetic Inhibition of CeA^GABA^ Neurons Alleviates DON‐induced Anorexia and Aversive‐like Emotions A) Virus (AAV9‐Hm4Di‐mCherry) injection in Vgat‐cre mice's CeA nucleus. B) Experimental Design and Timeline in DON condition. C) Traces of calcium fluorescence dynamics following CNO injection (lake‐blue trace for pre and rose‐pink trace for post) in CeA under DON condition. D) Heatmap visualized calcium fluorescence signal changes in CeA (n = 6 mice per group). E) Statistics of fluorescent calcium signal changes in pre and post CNO (n = 6 mice per group). F) Food Intake Post‐CNO Administration Schematic. G) Body Weight Changes in 24 h. H) Body Weight Changes in 4 h. I) Body Weight Changes in 24 h. J) OPT Motion Trajectory. K) OPT Total Distance. L) OPT Average Speed. M) OPT Center Duration. N) OPT Center Distance. O) EPM Motion Trajectory. P) EPM Open Arm Duration. Q) EPM Open Arm Entry. R) EPM Close Arm Duration. S) EPM Close Arm Entry. T) CPA Motion Trajectory. U) CPA Pre‐Test. V) CPA Post‐Test. n = 10 mice per group G‐V). Statistical Methods: Employed one‐way ANOVA (G, I, K‐N, P‐S, U‐V), two‐way ANOVA (H), and Welch‐corrected two‐tailed unpaired t‐test E) for data analysis. Note: Above all experiments were conducted under DON conditions.

Conversely, CeA^GABA^ neuronal inhibition was achieved through bilateral infection with AAV9‐DIO‐hM4Di‐mCherry (**Figure** **4**A; Figure S6, Supporting Information). Post‐CNO administration, calcium imaging under DON treatment revealed significantly decreased basal firing rates of GABA neurons (Figure 4B‐E), indicating effective chemogenetic deactivation. This resulted in an apparent trend in a reversal and recovery of body weight (Figure 3F‐G), but a statistically insignificant increase in food intake (Figure 4H‐I). Behavioral assessments (Figure 4J‐V) post‐DON treatment showed no significant differences in total movement, average speed, or exploration time in the OFT for hM4Di mice injected with CNO, but an increased distance traveled in the center area was observed (Figure 4K‐N). This resulted in a reversal and recovery of body weight (Figure 3F‐G) and an apparent trend, but a statistically insignificant, increase in food intake (Figure 4H‐I). Behavioral assessments (Figure 4J‐V) post‐DON exposure showed no significant differences in total movement, average speed, or exploration time in the OFT for hM4Di mice injected with CNO, but an increased distance traveled in the center area was observed (Figure 4K‐N). Additionally, CNO‐mediated deactivation led to increased time spent and entries into the open arms of the EPM (Figure 4P‐Q), while reducing the duration and entries into closed arms (Figure 4R‐S). CPA testing further confirmed a significant attenuation of aversive in the hM4Di group receiving CNO (Figure 4T‐V).

The above results comprehensively demonstrate that chemogenetic activation of CeA^GABA^ neurons mimics DON‐induced anorexia and aversive‐like emotions, while their inhibition facilitates recovery, thus illustrating their critical role in these behavioral phenomena.

### 2.4 Optogenetic Activation and Inactivation Experiments Further Validate the Behavioral Function of the CeA

Chemogenetic activation and inactivation confirmed that activation of CeA^GABA^ neurons induces anorexia and aversive‐like emotions, while their suppression reverses these effects. Optogenetic investigations further clarified whether these outcomes directly result from alterations in CeA neuronal activity, providing more details for understanding of the underlying mechanisms.

**Figure 5:**
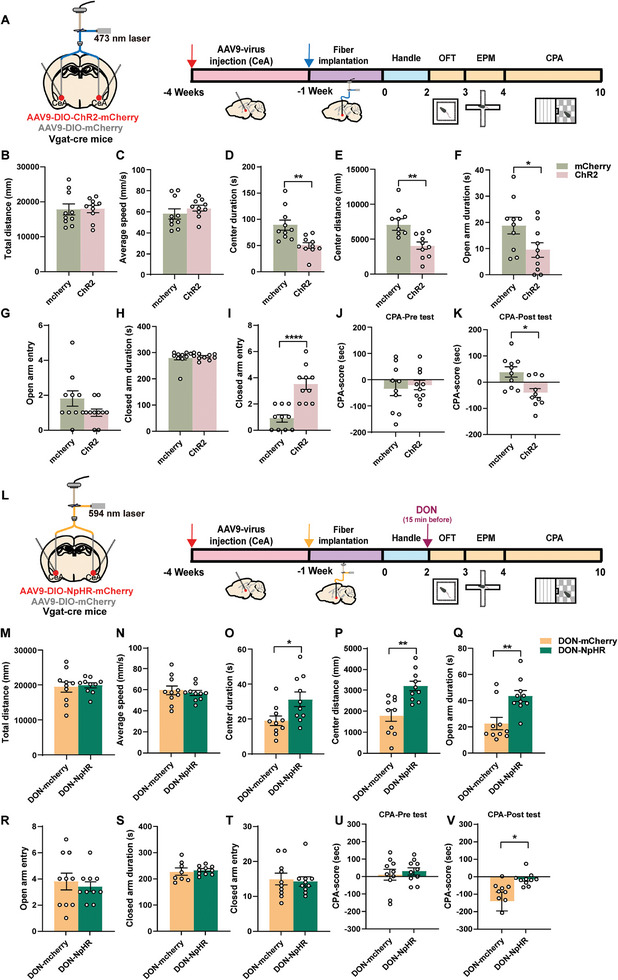
Optogenetic Regulation of CeA^GABA^ on DON‐induced Anorexia and Aversive‐like Emotions A) Virus (AAV9‐DIO‐ChR2‐mCherry) injection in Vgat‐cre mice's CeA nucleus and Control Virus Injection on the left. Experimental timeline depicted on the right. B) Total Distance in OPT: mCherry versus ChR2. C) Average Speed in OPT. D) Center Duration in OPT. E) Center Distance in OPT (P = 0.0027). F) Open Arm Duration in EPM. G) Open Arm Entry in EPM. H) Close Arm Duration in EPM. I) Close Arm Entry in EPM. J) Pre‐Test in CPA. K) Post‐Test in CPA. L) Virus (AAV9‐DIO‐NpHR‐mCherry) injection in Vgat‐cre mice's CeA nucleus and Control Virus Injection on the left. Experimental design shown on the right. M) Total Distance. N) Average Speed. O) Center Duration. P) Center Distance: Significant difference. Q) Open Arm Entry. R) Close Arm Duration. S) Close Arm Entry. T) Open Arm Duration. U) Pre‐Test in CPA. V) Post‐Test in CPA. n = 10 mice per group. Statistical Methods: Employed Welch‐corrected two‐tailed unpaired t‐test for all data analysis. L‐U experiments were conducted under DON conditions. *p* values calculated relative to Vehicle group.

In Vgat‐cre mice, bilateral infection of AAV9‐DIO‐ChR2‐mCherry in the CeA (**Figure** **5**A) was followed by inserting optical fibers at an inclined angle in the CeA with three weeks’ post‐stereotaxic surgery and one week of recovery. Blue light stimulation in mice expressing ChR2 proteins in the CeA activated GABA neurons, whereas it had no effect on neurons expressing mCherry. During the OPT process, activation of CeA^GABA^ neurons marked by ChR2 compared to the mCherry group showed no difference in total movement distance (Figure 5B) and average speed (Figure 5C) but a significant reduction in exploration time (Figure 5D) and distance in the center area (Figure 5E) (center distance: P = 0.0066; center duration: P = 0.0027). Similarly, in the EPM, blue light activation of ChR2‐marked neurons decreased the time spent in open arms (Figure 5F) and significantly increased entries into closed arms (Figure 5I). No differences were observed in entries into open (Figure 5G) or closed arms (Figure 5H). Additionally, in the CPA test (Figure 5J‐K), where mice received blue light stimulation upon entering the light‐stimulated room, thereby activating CeA^GABA^ neurons, ChR2 mice exhibited significantly less preference change compared to mCherry mice (Figure 5K), indicating aversive to the blue light room.

Subsequently, bilateral infection with AAV9‐DIO‐NpHR‐mCherry in the CeA of Vgat‐cre mice (Figure 5L) was performed, followed by similar post‐surgery procedures. Yellow light stimulation inhibited GABA neurons in the CeA of mice expressing NpHR proteins, but had no effect on neurons expressing mCherry. Under DON treatment, activation of CeA^GABA^ neurons marked by NpHR, compared to the mCherry group, showed no difference in total movement distance (Figure 5M) and average speed (Figure 5N), but a significant increase in exploration time (Figure 5O) and distance in the center area (Figure 5P) (center distance: *p* = 0.0011; center duration: *p* = 0.0219). Similarly, in the EPM (Figure 5Q‐T), yellow light activation of NpHR‐marked neurons increased the time spent in open arms (*p* = 0.0031, Figure 5Q). In the CPA test (Figure 5U‐V), mice received yellow light stimulation upon entering the light‐stimulated room, thereby inhibiting CeA^GABA^ neurons, NpHR mice exhibited reduced exposure to the yellow light room and alleviated DON‐induced aversive behavior.

### 2.5 Key Mediation of Anorexia and Aversive‐like Emotions by PVT‐CeA Circuit

PVT is intricately linked with the regulation of food intake and energy balance, playing a pivotal role in emotional responses.^[^ ^36^, ^37^ ^]^ Glutamate neurons in the PVT transmit excitatory signals that significantly influence these behaviors, especially in the context of anorexia and emotional regulation.^[^ ^21^, ^38^, ^39^ ^]^ Calcium/Calmodulin‐dependent protein kinase II (CaMKII) in the PVT modulates glutamate neuron activity.^[^ ^40^, ^41^, ^42^ ^]^ Activated by calcium influx, CaMKII exerts an impact on synaptic plasticity and neuron excitability in the PVT, thereby influencing feeding behaviors and emotional responses.^[^ ^27^, ^43^, ^44^ ^]^

**Figure 6:**
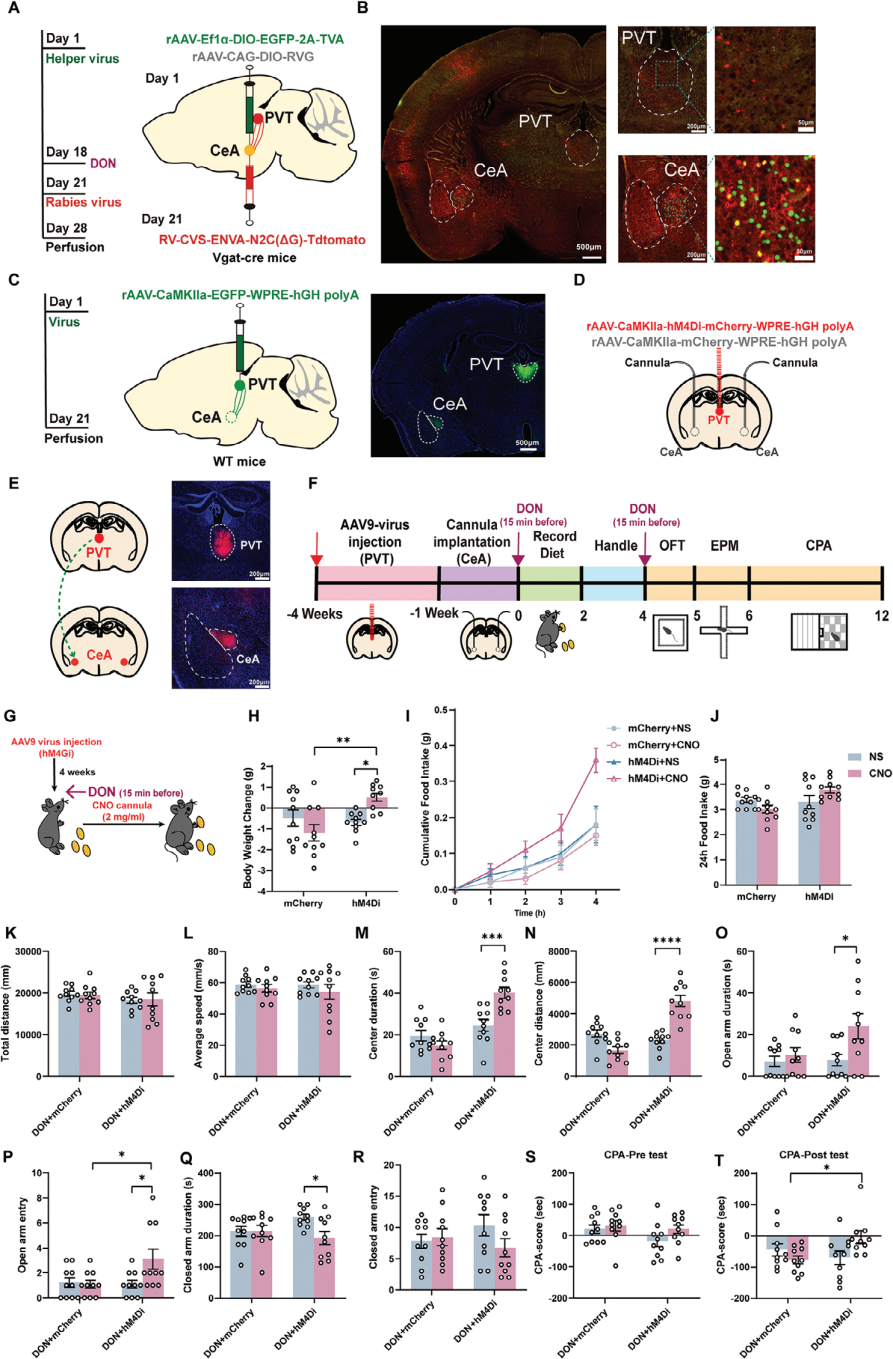
PVT‐CeA Projection and Chemogenetic Regulation Under DON Conditions A) Retrograde Tracing Schematic: Illustrates the tracing methodology. B) Fluorescent Expression Mapping: Tdtomato‐labeled PVT neurons projecting onto CeA visualized. Scale bars indicate 500 microns (left), 200 microns (middle), and 50 microns (right). C) Anterograde Tracing Schematic: Illustrates the tracing methodology (left) with corresponding fluorescence expression (right). Scale bar set at 500 microns. D) Chemogenetic Approach: Diagram detailing the viral and cannula embedded injection in the PVT‐CeA circuit. E) AAV9‐Hm4Di‐mCherry virus and control virus injection into PVT nucleus and embed the cannula in CeA 3 weeks later in WT mice. F) Experimental schemes and timeline in DON condition. G) Food Intake Post‐CNO Administration Schematic. H) Body Weight Changes in 24 h. I) Body Weight Changes in 4 h. J) Body Weight Changes in 24 h. K) OPT Total Distance. L) OPT Average Speed. M) OPT Center Duration. (N) OPT Center Distance. O) EPM Open Arm Duration. P) EPM Open Arm Entry. Q) EPM Close Arm Duration. R) EPM Close Arm Entry. S) CPA Pre‐Test. T) CPA Post‐Test. N = 10 mice per group. Statistical Methods: Employed one‐way ANOVA (H, J‐T), and two‐way ANOVA (I) for data analysis. Above all experiments were conducted under DON conditions.

We first investigated the anatomical characteristics of PVT to CeA projections using retrograde and anterograde tracing (**Figure** **6**A‐C). To retrogradely trace neurons projecting to the CeA from the PVT, Cre‐dependent auxiliary viruses (rAAV‐Ef1α‐DIO‐EGFP‐2A‐TVA and rAAV‐CAG‐DIO‐RVG) were injected into the CeA, followed by rabies virus (RV‐CVS‐ENVA‐^Δ^G‐Tdtomato) within three weeks later (Figure 6A). The auxiliary viruses facilitated monosynaptic retrograde transport of the rabies virus, allowing us to trace projections to the CeA. We observed a significant increase in Tdtomato‐labeled PVT neurons projecting to the CeA (Figure 6B; Figure S7A‐C, Supporting Information), accompanied by enhanced synaptic neurotransmitter release, as indicated by the decreased paired‐pulse ratio (PPR) in the PVT‐CeA pathway (Figure S7D‐E, Supporting Information). Next, an adeno‐associated virus encoding the enhanced green fluorescent protein (rAAV‐CaMKIIα‐EGFP) was injected into the PVT for anterograde tracing of axonal terminals in the CeA, revealing dense EGFP‐positive axonal endings (Figure 6C).

To investigate the causal relationship of the PVT‐CeA circuit in anorexia and aversive, chemogenetics was employed to assess its impact on feeding and emotional responses. Post three‐week injection of rAAV‐CaMKIIα‐hM4Di‐mCherry virus into the PVT of wild‐type (WT) mice, cannulas were angled into the CeA for CNO administration, allowing a week's recovery (Figure 6D‐F). Schematic representation of the PVT and CeA areas are depicted in Figure 6E. Under DON treatment, CNO activation in hM4Di mice reversed weight loss (mCherry+CNO versus hM4Di+CNO: *p* = 0.0016; hM4Di+NS versus hM4Di+CNO: *p* = 0.0404, Figure 6G‐H) and exhibited a notable, though not statistically significant, increase in food intake (Figure 6I‐J). In parallel, DON‐mimicking experiments activating CeA^GABA^ neurons in the CeA (Figure S8A, Supporting Information) showed substantial weight decrease in hM3Dq mice post CNO activation (Figure S8B‐C, Supporting Information) with similar trends in food consumption (Figure S3D‐E, Supporting Information).

Behaviorally, CNO‐treated hM4Di mice demonstrated a significant elevation in exploration time (DON+hM4Di+NS versus DON+hM4Di+CNO: *p* = 0.0005, Figure 6M) and distance in the central area of the OPT (DON+hM4Di+NS versus DON+hM4Di+CNO: *p* < 0.0001, Figure 6N), with no change in total movement or average speed (Figure 6K,L). This result indicates a partial mitigation of DON induced negative behaviors by hM4Di. In contrast, DON‐mimicking experiments showed opposite effects (Figure S8F‐I, Supporting Information). In EPM, CNO‐induced deactivation of CeA^GABA^ neurons led to increased time and entries in open arms (DON+hM4Di+NS versus DON+hM4Di+CNO: *p* = 0.0278, Figure 4O; *p* = 0.0171, Figure 6P) and a reduction in time spent in closed arms (DON+hM4Di+NS versus DON+hM4Di+CNO: *p* = 0.0381, Figure 4Q), with a trend toward fewer entries (Figure 6R). Conversely, the mimicking experiments displayed strong aversive (Figure S8N‐O, Supporting Information), with a significant decrease in time spent in the DON‐treatment room (DON+hM4Di+NS versus DON+hM4Di+CNO: *p* = 0.0045, Figure S8O, Supporting Information). In CPA test, all groups except the CNO‐treated hM4Di mice showed strong aversive emotions (Figure 6S‐T), whereas aversive was notably reduced in the hM4Di+CNO group, evidenced by increased time in DON‐treatment room (DON+mCherry+CNO versus DON+hM4Di+CNO: *p* = 0.043, Figure 6T).

### 2.6 Optogenetic Further Validation of the Role of the PVT‐CeA Circuit in DON‐Induced Aversive Behaviors

**Figure 7:**
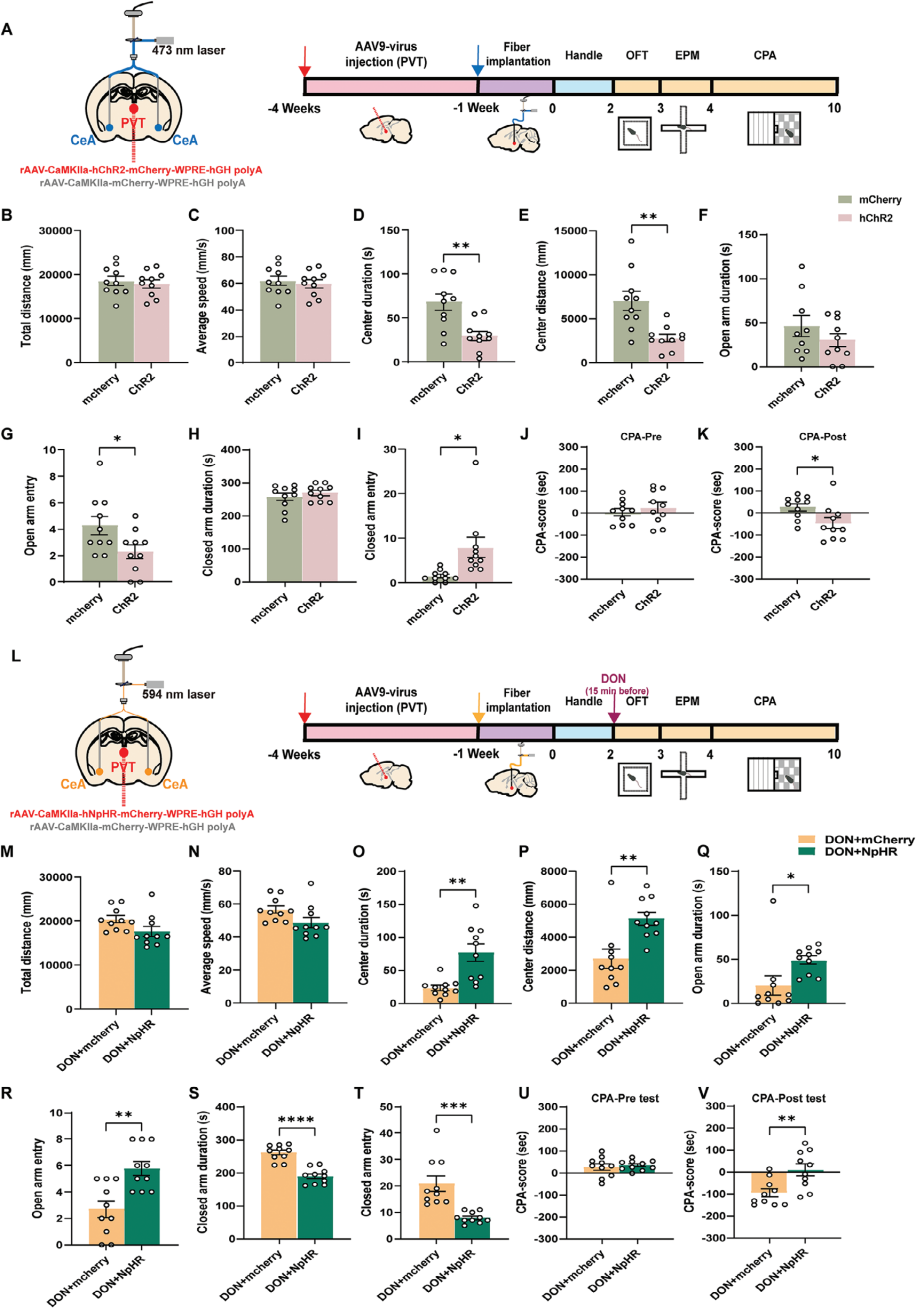
Optogenetic Experiment Testified Regulation of PVT‐CeA Circuit for behaviors. A) Experimental Schematic: AAV9‐DIO‐ChR2‐mCherry and control virus AAV9‐DIO‐mCherry injection into PVT nucleus in WT mice, followed by fiber embedding in CeA after 3 weeks. Right image details experimental timeline. B) Total Distance in OPT. C) Average Speed in OPT. D) Center Duration in OPT E) Center Distance in OPT. F) Open Arm Duration in EPM. G) Open Arm Entry in EPM. H) Close Arm Duration in EPM. I) Close Arm Entry in EPM. J) Pre‐Test in CPA. K) Post‐Test in CPA. L) Experimental Schematic: AAV9‐DIO‐NpHR‐mCherry and control virus injection into PVT nucleus with subsequent fiber embedding in CeA in WT mice e under DON conditions. Right image presents experimental timeline. M) Total Distance in OPT. N) Average Speed in OPT. O) Center Duration in OPT. P) Center Distance in OPT. Q) Open Arm Duration: Significant difference in EPM. R) Open Arm Entry in EPM. S) Close Arm Duration in EPM. T) Close Arm Entry in EPM. U) Pre‐Test in CPA. V) Post‐Test in CPA. n = 10 mice per group. Statistical Methods: Employed Welch‐corrected two‐tailed unpaired t‐test for all data analysis. L‐V experiments were conducted under DON conditions. *p* values calculated relative to vehicle group.

We infected the PVT with rAAV‐CaMKIIα‐hChR2‐mCherry for three weeks from stereotaxic surgery in WT mice, optical fibers were inserted into the CeA at an inclined angle, followed by a week of recovery (**Figure** **7**A). The CeA receiving neurons expressing ChR2 from the PVT were stimulated with blue light to mimic DON treatment. Similarly, AAV‐CaMKIIα‐hNpHR‐mCherry was infused, and yellow light stimulation in the CeA was used for DON reversal experiments (Figure 7L). Results indicated that activation of ChR2‐expressing axonal terminals projecting from the PVT to CeA did not alter total movement distance or average speed (Figure 7B‐C), but significantly reduced exploration time and distance in the center area of the OPT (*p* = 0.0022 and *p* = 0.0019, respectively, Figure 7D‐E). In EPM, a significant decrease in entries into open arms (Figure 7G) and an increase in closed arm entries (Figure 7I) were observed, with no marked differences in time spent in open (Figure 7F) or closed arms (Figure 7H). Additionally, in CPA test (Figure 7J‐K), ChR2 mice exhibited strong aversive to the blue light‐stimulated room (*p* = 0.0249, Figure 7K).

Conversely, the inhibition of axonal terminals projecting from the PVT to CeA, marked with NpHR, led to a significant increase in exploration time and distance in the center area of the OPT (*p* = 0.0011 and *p* = 0.0023, respectively, Figure 7O‐P), with no changes in total movement distance (Figure 7M) or average speed (Figure 7N). In the EPM, this resulted in increased time spent and entries into open arms (*p* = 0.0276; *p* = 0.0015, respectively, Figure 7Q‐R) and decreased time and entries into closed arms (*p* < 0.0001; *p* = 0.0004, Figure 7S‐T). In the CPA test (Figure 7U‐V), aversive behavior tendency of the yellow light‐stimulated room was alleviated in NpHR mice (*p* = 0.0048, Figure 7V). These findings further reveal the critical role of CeA^GABA^ neuronal activity in regulating anorexia and aversive emotions, with distinct behavioral impacts noted upon their activation and inhibition.

### 2.7 A‐803467 Rescue DON‐Induced Anorexia and Aversive‐Like Emotions

To further explore the role of PVT‐CeA circuit in anorexia and aversive in mice, transcriptomic sequencing of CeA area is necessary. It is essential to elucidate the dynamics of gene expression within the CeA, particularly those genes that fluctuate during feeding and emotional regulation processes. By analyzing these gene expression data, we can identify critical molecular targets associated with anorexia and aversive‐like emotions.

**Figure 8:**
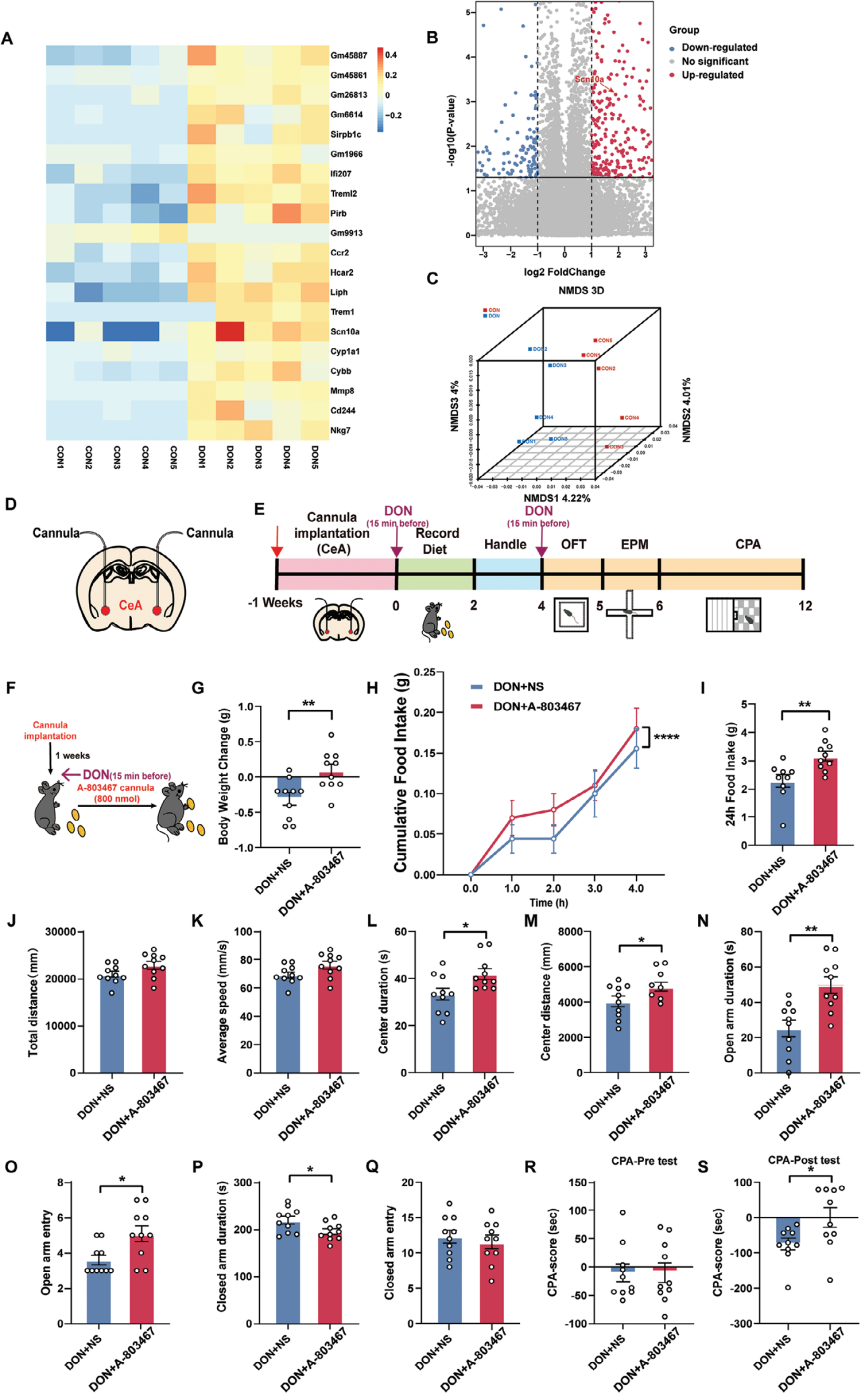
A‐803467 Rescue DON‐induced Anorexia and Aversive‐like Emotions A) Heatmap depicting genes the significance levels and corresponding changes in CeA. Values were normalized to the base mean for each gene; n = 5 mice per group. B) Volcano plot depicting differentially expressed genes in CeA neurons treated with CON or DON for 15 min. Dashed lines reveal fold change and significance thresholds. Up‐regulated and down‐regulated genes are shown in red and blue, respectively. C) Non‐metric Multidimensional Scaling (NMDS) analysis chart. Points of different colors or shapes represent sample groups under different environments or conditions, and the scales of the horizontal and vertical coordinates are relative distances. D) Cannula implantation: cannula was embedded in CeA of WT mice. E) Experimental schemes and timelines. F) Food Intake after A‐803467 Administration Schematic. G) Body Weight Changes in 24 h. H) Body Weight Changes in 4 h. I) Body Weight Changes in 24 h. J) OPT Total Distance. K) OPT Average Speed. L) OPT Center Duration. M) OPT Center Distance. N) EPM Open Arm Duration. O) EPM Open Arm Entry. P) EPM Close Arm Duration. Q) EPM Close Arm Entry. R) CPA Pre‐Test. S) CPA Post‐Test. N = 10 mice per group. Statistical Methods: Employed two‐way ANOVA H) and Welch‐corrected two‐tailed unpaired t‐test (G, I‐S) for data analysis.

We first sequenced mRNA expression in the CeA area, presenting the top 20 regulated mRNA levels in a heatmap, with each mRNA value normalized to a baseline average (**Figure** **8**A). Subsequently, volcano plot analysis revealed differentially expressed genes in the CeA area post NS and DON treatment (Figure 8B). Furthermore, we conducted a non‐metric multidimensional scaling analysis (NMDS) to visually demonstrate the differences between the CON and DON groups (Figure 8C). We found a significant increase expression of the gene encoding the NaV1.8 voltage‐gated sodium ion channel (SCN10A), primarily expressed in sensory neurons. Whether inhibiting SCN10A expression could rescue DON‐induced anorexia and aversive, we implanted cannulas bilaterally in the CeA area for administration of the SCN10A antagonist (A‐803467) (Figure 8D‐E). We found that compared to the DON group, mice treated with A‐803467 reversed weight loss (*p* = 0.0054, Figure 8F‐G) and significantly reversed food intake at the first 4 h and at 24 h (*p* < 0.0001 and *p* = 0.0055, respectively, Figure 8H‐I).

Next, in OPT experiment, we observed no significant changes in total movement distance and average speed (Figure 8J‐K), but a notable reduction in exploration time and distance in the center area (*p* = 0.0202, *p* = 0.047, respectively, Figure 8L‐M). In EPM, time spent and entries into open arms increased (*p* = 0.0015, *p* = 0.0111, respectively, Figure 8N‐O), while no significant changes were observed in closed arm duration and entries (Figure 8P‐Q). In CPA test, mice treated with A‐803467 showed a significant alleviation of aversive to the DON‐treatment room (*p* = 0.0289, Figure 8R‐S). Further supporting these behavioral findings, we found that administration of the SCN10A antagonist (A‐803467) suppressed the abnormal increase in action potential firing induced by DON activation, thereby significantly reducing neuronal activity in the CeA (Figure S9, Supporting Information).

## 3 Discussion

Our study resolves a long‐standing toxicological conundrum by elucidating the neural circuitry that regulate DON‐induced anorexia and aversive‐like emotions. We emphasize the significance of delving into the modulation of GABA neurons in the PVT‐CeA circuit. Our observations revealed that acute administration of DON in mice precipitated substantial declines in various physiological and behavioral parameters including food intake, locomotor activity, HEAT response, and feeding frequency. Simultaneously, there were significant behavioral changes noted in the OPT, EPM, and CPA tests. Intriguingly, different doses of DON (2.5 and 5 mg kg^−1^) were found to diminish the activity of theta and alpha brain waves. Subsequent exploration highlighted an intensified activation of CeA^GABA^ neurons after DON exposure, a phenomenon that closely correlated with the development of anorexia and aversive effects. Manipulating GABA neurons in the PVT‐CeA circuit through chemogenetic and optogenetic approaches significantly altered the mice's anorexic and aversive effects. Additionally, transcriptomic sequencing revealed notable upregulation of the Scn10A in CeA, implicating its potential role in these behavioral manifestations. Utilizing the Scn10A antagonist, A‐803467, was observed to effectively counteract DON‐induced anorexia and aversive‐like emotions, offering a promising therapeutic pathway. Collectively, our study established an input‐output framework for the PVT‐CeA circuit's involvement in anorexia and aversive effects, enriching the comprehension of its functional intricacies in these contexts.

In previously published studies, Pestka and co‐authors^[^ ^45^, ^46^ ^]^ determined that the No Observed Adverse Effect Level (NOAEL) and the Lowest Observed Adverse Effect Level (LOAEL) for oral DON exposure were 1 or/and 2.5mg kg^−1^ bw, respectively. These studies, providing guidelines for DON dosages, are crucial for public health.^[^ ^47^, ^48^ ^]^ Our findings also indicate the varying impact of DON on food intake, behavior, and electrophysiology with different concentrations. Remarkably, a 2.5 mg kg^−1^ dose of DON induces anorexia and aversive‐like emotions without tissue pathological changes.^[^ ^49^, ^50^ ^]^ However, a higher dose of 5 mg kg^−1^ directly results in severe pathological damage, complicating behavioral interpretation under pathological states, while 1 mg kg^−1^ does not elicit notable changes in anorexia or emotions.^[^ ^51^ ^]^ From a public health perspective, the 2.5 mg kg^−1^ dose is compatible, serving as a critical warning threshold beyond which physical damage occurs.^[^ ^50^ ^]^ Therefore, we selected 2.5 mg kg^−1^ as the suitable dose for whole investigations. Given the higher susceptibility of female mice to anorexia, which mirrors the gender differences observed in humans,^[^ ^52^ ^]^ we focused on female mice in our study. It is supported by evidence that hormonal influences make female mice more responsive to anorexia models, allowing us to establish a robust framework for investigating DON‐induced effects.^[^ ^53^ ^]^

The PVT plays a crucial role in various brain functions, including reward, arousal, emotion, and behavioral regulation. It acts as a key neural hub, receiving inputs from the reticular formation and hypothalamus, and participating in diverse brain circuits.^[^ ^43^, ^54^, ^55^, ^56^, ^57^ ^]^ Neuroanatomical tracing and functional manipulation experiments highlight that Type I neurons in the posterior PVT (pPVT) are implicated in aversive states.^[^ ^38^, ^58^ ^]^ These neurons play a pivotal role in processing aversive stimuli, leading to emotional and behavioral responses such as fear, anxiety, or avoidance.^[^ ^56^, ^59^ ^]^ The CeA, a primary output area of the PVT, also regulates feeding, energy metabolism, and emotional responses.^[^ ^55^, ^60^, ^61^ ^]^ While the PVT‐CeA circuit has been also associated with anxiety and depression‐related behaviors, recent studies demonstrated its role in negative emotions, yet its simultaneous influence on anorexia and aversive emotions remains undocumented.^[^ ^62^, ^63^, ^64^, ^65^, ^66^, ^67^, ^68^ ^]^

In this study, the activation of CeA^GABA^ neurons led to a reduction in food intake, body weight and evoke aversive‐like emotions (Figures 3 and 5A‐K). These neurons, located in the central and dorsomedial CeA, primarily use GABA as a neurotransmitter. Our findings indicate that GABAergic projections from the PVT to the CeA contribute to feeding suppression and aversive emotions (Figure S2, Supporting Information; Figure 7A‐K). Conversely, inhibiting these GABA neurons, whether in the PVT or their projections to the CeA, reverses anorexia and alleviates aversive (Figures 4, 5,6). The PVT receives GABAergic inputs from the zona incerta (ZI), a brain area involved in defensive and fear behaviors. Activation of ZI^GABA^ inputs to the PVT induces binge eating behaviors.^[^ ^69^ ^]^ However, our results demonstrate that PVT project excitatory inputs to the CeA^GABA^ neurons, resulting in reduced food intake and the induction of aversive responses. Additionally, ZI^DA^ neurons, activated by environmental food cues, primarily regulate fasting‐induced food seeking.^[^ ^69^ ^]^ Our observations suggest that inhibiting GABA neurons in the CeA under DON conditions reduces excitation in specific pathways, thereby alleviating anorexia and aversive‐like behaviors. Previous studies have associated the CeA to negative emotional behaviors and demonstrated that PVT‐CeA projections play a crucial role in aversive memory formation.^[^ ^67^, ^70^ ^]^

In conclusion, our research has unveiled the previously unexplored function of the PVT‐CeA circuit in modulating DON‐induced anorexia and aversive emotions. Inhibiting GABAergic neurons activity in this circuit alleviates these phenomena. These findings lay the foundation for future investigations to delineate the specific contributions of this neural circuit in mediating anorexic and aversive effects responses to DON and other conditions.

## 4 Experimental Section

### Chemicals

DON (12, 13‐epoxy‐3, 4, 15‐trihydroxytrichotec‐9‐en‐8‐one, C_15_H_20_O_6_, MW: 296.32, purity ≥ 99%, CAS number: 51481‐10‐8) was purchased from FERMENTEK. Ltd (Item No. MSS1011, Jerusalem, Israel). DON solution for gavage was fully dissolved in 0.9% normal saline.

### Animals

Female wild‐type C57BL/6J mice (8 weeks old, 25–30 g) were obtained from Vital River Laboratory Animal Technology Co., Ltd (Beijing, China). Vgat‐ires‐cre STOCK Slc32a1^tm2(cre)Lowl^/J mice (016962) were obtained from Jackson Lab (CA, USA), and were bred on a C57BL/6J background for more than six generations. Female offspring (aged 6–8 weeks) were used in the experiments and were allowed to adapt to the housing in the specific pathogen‐free (SPF) system room (Pesticide Toxicology Center, Tongji Medical College, Huazhong University of Science and Technology) for one week prior to random assignment into experimental and vehicle cohorts. Otherwise, we randomly assigned each mouse to every group with 8 or 10 mice in each group using a random number table, called complete randomization. The mice were housed under standard conditions with controlled humidity (55 ± 5%), temperature (22 ± 2 °C), ventilation (air exchange rate of 18 times per hour), 12 h light/dark cycle, and fed a standard rodent laboratory diet (Altromin 1324, Brogaarden, Denmark) and sterile tap water freely. All experiments were approved by the Animal Care and Use Committee of Tongji Medical College, Huazhong University of Science and Technology (IACUC Number: 3574) and were performed by Animal Research Institute Committee guidelines (Guide for the Care and Use of Laboratory Animals, National Institute of Health, 1996, Bethesda, MD, USA).

### Food Intake Measurement

Food and water intake, oxygen and carbon dioxide consumption, heat production, respiratory exchange rate, and ambient temperature and humidity were quantitatively assessed using the Oxymax/CLAMS Small Animal Metabolic System (Columbus Instruments, USA).

### Virus Preparations

We used viruses packaged and obtained by BrainVTA (Wuhan, China): rAAV‐Ef1α‐DIO‐EYFP‐WPRE‐hGH polyA (Cat. No. PT‐0012), rAAV‐hSyn‐DIO‐hM3D(Gq)‐mCherry‐WPRE‐hGH polyA (Cat. No. PT‐0019), rAAV‐hSyn‐DIO‐hM4D(Gi)‐mCherry‐WPRE‐hGH polyA (Cat. No. PT‐0020), rAAV‐EF1α‐DIO‐hChR2(H134R)‐mCherry‐WPRE‐hGH polyA (Cat. No. PT‐0002), rAAV‐EF1α‐DIO‐eNpHR3.0‐mCherry‐WPRE‐hGH polyA (Cat. No. PT‐0007), rAAV‐Ef1α‐DIO‐mCherry‐WPRE‐hGH polyA (Cat. No. PT‐0013), rAAV‐CaMKIIa‐hM3D(Gq)‐mCherry‐WPREs (Cat. No. PT‐0013), rAAV‐CaMKIIa‐hM4D(Gi)‐mCherry‐WPRE‐hGH polyA (Cat. No. PT‐0017), rAAV‐CaMKIIa‐hChR2(H134R)‐mCherry‐WPRE‐hGH polyA (Cat. No. PT‐0297), rAAV‐CaMKIIa‐eNpHR3.0‐mCherry‐WPRE‐hGH polyA (Cat. No. PT‐0009), rAAV‐CaMKIIa‐mCherry‐WPRE‐hGH polyA (Cat. No. PT‐0108), rAAV‐EF1α‐DIO‐GCaMp6m‐WPRE‐hGH polyA (Cat. No. PT‐0283), rAAV‐Ef1α‐DIO‐EGFP‐2A‐TVA ‐WPRE‐hGH polyA (Cat. No. PT‐0062), rAAV‐CAG‐DIO‐RVG‐WPRE‐hGH polyA (Cat. No. PT‐0519), and rAAV‐CaMKlla‐EGFP‐WPRE‐hGH polyA (Cat. No. PT‐0290). The above viral titers at 5.0 × 10^12^ vg/mL, and RV‐CVS‐ENVA‐N2C(^Δ^G)‐tdtomato (Cat. No. R05002), viral titer at 3 × 10^8^ IFU/mL. Divide the viral vector into aliquots and store at −80 °C until use.

### Behavioral Testing

One week before the start of the experiment, the mice were gently stroked every day to familiarize the mice with the smell of the experimental operator. On the day of the test, the mice were moved to the testing room and adapted to the room conditions for at least 1 hour. After each individual test, clean the equipment thoroughly with 75% alcohol to eliminate odors and traces of previously tested mice. The time point of each experiment was fixed at black cycles (18:00‐6:00) to avoid affecting the basal exercise level of mice due to different experimental time points and causing deviation in experimental results.

*Open Field Testing (OFT)*: Using an activity monitoring system (50 cm long × 50 cm wide × 45 cm high, SuperMaze, Xinruan, Shanghai, China) for detecting locomotor activity. Each mouse place in the center of the open field and allow the mice to freely explore the surrounding environment. The open space is divided into central and peripheral areas. A video tracking system (SuperMaze, Xinruan, Shanghai, China) was used to measure the spontaneous activity of mice. Divide the area into 25 equal squares and take the middle 9 squares as the central area. The center is defined as a square accounting for 36% of the total OFT area. Monitoring records and analyzes the time in the central area and the total distance traveled, the performs a 5‐minute statistical analysis of its behavior.

*Elevated Plus Maze Test (EPM)*: The elevated maze consisted of four arms (length 35 cm × width 5 cm × height 15 cm) and a central platform located 75 cm above the floor. Two of the arms have 15 cm high dark walls (closed arms) and two arms have 0.5 cm high ledges (open arms). The arms are at a 90° angle to each other. The device was placed in a quiet and dimly lit room. The mice were placed on the central platform to monitor their behavior with a camera (SuperMaze, Xinruan, Shanghai, China) placed on the top of the maze, and the behavior was statistically analyzed for 5 min.

*Conditioned Place Avoidance (CPA)*: Mice were placed in boxes of equal size in terms of length, width, and height (20 × 20 × 35 cm^3^) on both sides, with each side measuring 30 × 25 cm^2^. The experiment was conducted at room temperature (23 °C). One side of the room had black and white alternating stripe patterns, while the other side featured a black and white checkerboard pattern, with equal areas of black and white. In the preliminary phase before formal training, the partitions between the chambers were removed, and the experimental animals were placed in the experimental boxes to allow them to move freely. The time spent by the animals in each box was recorded. This phase aimed to familiarize the animals with the experimental apparatus, reduce novelty and stress, and assess non‐conditioned preferences to select the animals. This phase lasted for a minimum of 3 days, with daily sessions lasting 30 min each. On the first day of testing, baseline measurements were obtained as the mice freely explored the boxes. The process of mouse exploration was recorded using cameras placed on top of the maze (SuperMaze, Shanghai, China). Each mouse explored for 30 min.

During the training phase, partitions segregated the rooms, confining animals to a designated room for 30 min before removal. Control experiments were concurrently conducted either with a 4–8 h interval on the same day or at an identical time on a subsequent day.

During the training phase, partitions isolated animals within designated rooms for 30 min before their removal. Control experiments were either interspersed with 4–8 h gaps on the same day or scheduled at the same time on the following day. For training in DON‐induced aversive‐like emotion, mice underwent a simulated learning process in a specific room area, commencing 15 min after DON gavage. In optogenetic simulation experiments, mice experienced 20 Hz blue light stimulation (470 nm, 15 ms pulse, 20 Hz, 10 mW), designed to replicate DON's effects and activate GABAergic neurons, following a pattern of 10 s on and 10 seconds off. The chemogenetic approach involved intraperitoneal injections of Clozapine N‐oxide (CNO) or saline prior to the start of the experiment. During chemical/optogenetic reversal training, the chemogenetic group received intraperitoneal injections of CNO or saline 15 min after DON gavage before being placed in a designated section of the room, whereas the optogenetic reversal group underwent yellow light stimulation.

Subsequent to the training phase, experimental animals underwent testing. During these tests, no drug treatments were administered, and animals were allowed free movement between chambers in a corridor with partitions removed.

### Surgical Electroencephalogram (EEG) Electrode Placement and Recording

Mice were anesthetized using ketamine (100 mg kg^−1^, i.p.) and dexmedetomidine (0.5 mg kg^−1^, i.p.) and placed in a stereotaxic apparatus (RWD, Shenzhen, China), with a heating pad maintaining stable body temperature. Subcutaneous bupivacaine was administered for analgesia, followed by the surgical implantation of extradural EEG electrodes. Two stainless steel screws were positioned in the right frontal cortex (0.4 mm lateral, 1.75 mm anterior to bregma) and the left cerebellum for EEG recording.^[^ ^71^, ^72^ ^]^ Leads were connected to a miniature plug and affixed to the skull with Super‐Bond C&B and dental acrylic.^[^ ^73^ ^]^ Postoperatively, mice recovered on a heated sheet and acclimatized to a standard light‐dark cycle for 7 days before recording. Mice were tethered to a rotative connector via a cable for unrestricted movement in cylindrical chambers. EEG recordings were executed at a 500 Hz sampling frequency using a Model 1700 differential AC amplifier (A‐M System, Carlsborg, WA, USA) and a PCIe 6323 data acquisition board (National Instruments, Austin, TX, USA). Continuous EEG signal acquisition was performed with Spikehound software.

### c‐fos Immunofluorescence and Quantification

Following oral administration of DON (2.5 mg kg^−1^, 90 min) or normal saline, mice were anesthetized by ketamine (100 mg kg^−1^, i.p.) and dexmedetomidine (0.5 mg kg^−1^, i.p.) and perfused apically first with 0.9% normal saline, then with 4% paraformaldehyde (PFA). The brain tissues were extracted, dehydrated via a 20–30% sucrose gradient, and sectioned at 30 µm using a Leica CM9800 cryostat for c‐fos immunohistochemistry. Free‐floating sections were incubated in primary antibody (rabbit anti‐c‐Fos, 1:500, Cell Signaling Technology, Item No: 2250S, China) at 4 °C for 48 h, washed three times with PBS, followed by incubation with CoraLite 488 conjugated goat anti‐rabbit IgG secondary antibody (SA00013‐2, Proteintech Group, Inc., China) at room temperature for 2 h. After PBS washed and mounted with anti‐fluorescence quenching solution. Fluorescence images were captured using a VS120 Olympus automatic section scanning system (10× objective lens, Japan) and analyzed statistically with Image J software.

### Electrophysiological Recordings

Mice were anesthetized with ketamine (100 mg kg^−1^, i.p.) and dexmedetomidine (0.5 mg kg^−1^, i.p.), then perfused with 40 mL ice‐cold slicing solution (210 mM sucrose, 3.1 mM sodium pyruvate, 11.6 mM sodium L‐ascorbate, 1.0 mM NaH_2_PO_4_, 26.2 mM NaHCO_3_, 5.0 mM MgCl_2_, 20.0 mM glucose; pH 7.4). CeA regions were sectioned coronally at 300 µm and incubated in aCSF (128.0 mM NaCl, 3.0 mM KCl, 24.0 mM NaHCO_3_, 2.0 mM MgCl_2_, 1.25 mM Na_2_HPO_4_, 10.0 mM D‐glucose, 2.0 mM CaCl_2_; 95% O_2_/5% CO_2_, pH 7.2‐7.4, 295–305 mOsm) at 28 °C for 1 hour. For mIPSC recordings, slices were treated with tetrodotoxin (10 µM) and AMPA/kainate antagonist (10 µM); the electrode solution contained 153.3 mM CsCl, 1.0 mM MgCl_2_•6H_2_O, 5.0 mM EGTA, 4.0 mM Mg‐ATP, 10.0 mM HEPES (pH 7.25, 280–300 mOsm). For mEPSCs, tetrodotoxin (10 µM) and bicuculline (20 µM) were used, with an electrode solution of 122.5 mM CsMeSO_3_, 17.5 mM CsCl, 0.2 mM EGTA, 10.0 mM HEPES, 1.0 mM MgCl_2_, 4.0 mM Mg‐ATP, 0.3 mM Na‐GTP, 5.0 mM QX314 (pH 7.25, 280–300 mOsm). Recordings were made using a Multiclamp 700B amplifier.

### In Vivo Optic‐Fiber Recordings

Optical fibers (200 outer diameters, 0.37 NA, RWD, Shenzhen, China) were implanted into the CeA of mice. Fluorescence recording utilized a Fiber Photometry system with 470‐ and 410‐nm excitation lasers (RWD, Shenzhen, China). Recorded fluorescence signals, processed using a script from RWD, were normalized to baseline to calculate ∆F/F, defined as (F‐F0)/F0, where F0 represents the mean integral.^[^ ^74^, ^75^ ^]^

Data analysis incorporated baseline and motion‐correction strategies. The 410‐nm signal served as a reference to mitigate motion‐related artifacts. This signal was adjusted through least‐squares regression to align with the 470‐nm signal and then subtracted from it to yield the motion‐corrected 470‐nm signal.^[^ ^76^ ^]^ To counteract photo‐bleaching from prolonged recording, the polynomial fitted correction was applied. Analysis, conducted in MATLAB, presented ∆F/F values as heatmaps and average plots, with the shaded area representing standard error of the mean. Mice with inaccurate fiber‐tip placement were excluded from the analysis.

### Stereotactic Surgeries

For viral injection, mice were anesthetized using ketamine (100 mg kg^−1^) and dexmedetomidine (0.5 mg kg^−1^) and positioned in a stereotaxic device (RWD Life Technologies Co., Ltd., China). Regarding to procedure, conducted on a 37 °C heating pad, involved shaving the mouse's head, making a midline incision to expose the skull, and disinfecting with povidone‐iodine. The skull was aligned using the XYZ axis (0.03 mm error margin), and a small cranial opening was drilled above the injection site. The virus was administered into the target brain area via microinjection (20‐25 nL min^−1^), with the needle retained at the site for 10 minutes post‐injection. Mice were allowed a recovery period of 2 weeks before experimental procedures.

In whole‐cell patch‐clamp experiment, rAAV‐Ef1α‐DIO‐EYFP‐WPRE‐hGH polyA (150 nL) was injected into the CeA of Vgat‐cre mice. Electrophysiological recordings were conducted in the Vehicle group and DON group after three weeks.

In fiber photometry experiments, rAAV‐EF1α‐DIO‐GCaMp6m‐WPRE‐hGH polyA (150 nL) was injected into the CeA of Vgat‐cre mice. After 14 days, a ceramic optical fiber cannula (200 µm, 0.37 NA, RWD) was implanted above the CeA for data recording and analysis.

In chemogenetic stimulation experiments involving hM3Dq/hM4Di, Cre‐dependent hM3D virus (rAAV‐hSyn‐DIO‐hM3D(Gq)‐mCherry‐WPRE‐hGH polyA) and hM4Di virus (rAAV‐hSyn‐DIO‐hM4D(Gi)‐mCherry‐WPRE‐hGH polyA) were bilaterally injected into the CeA (AP: −0.95 mm; ML: ±2.65 mm; DV: −4.75 mm) or PVT region (AP: −1.10 mm; ML: ±0.50 mm; DV: −2.90 mm) at a 10° angle. The control virus (rAAV‐Ef1α‐DIO‐mCherry‐WPRE‐hGH polyA) and other chemogenetic vectors were administered at volumes of 150 nL (CeA) and 100 nL (PVT), at rates of 25 nL min^−1^ and 20 nL min^−1^, respectively. Post‐injection, a cannula (RWD, model: 62002, 24G) was implanted above the CeA for CNO injections (100 nL, 2 mg kg^−1^).

In optogenetic experiments for in situ activation, Cre‐dependent viruses (rAAV‐hSyn‐DIO‐hChR2(H134R)‐mCherry‐WPRE‐hGH polyA, rAAV‐hSyn‐DIO‐eNpHR3.0‐mCherry‐WPRE‐hGH polyA, and rAAV‐Ef1α‐DIO‐mCherry‐WPRE‐hGH polyA) were bilaterally injected into the CeA (AP: −0.95 mm; ML: ±2.65 mm; DV: −4.75 mm) at 150 nL. Following 21 days of viral expression, ceramic optical fiber cannulas (200 µm, 0.39NA, RWD) were implanted 3 mm above the CeA injection sites. The same protocol applied to the PVT‐CeA circuit with Cre‐dependent viruses (rAAV‐CaMKIIa‐hChR2(H134R)‐mCherry‐WPRE‐hGH polyA, rAAV‐CaMKIIa‐eNpHR3.0‐mCherry‐WPRE‐hGH polyA, and rAAV‐CaMKIIa‐mCherry‐WPRE‐hGH polyA) were bilaterally injected into the PVT region (AP: −1.10 mm; ML: ±0.50 mm; DV: −2.90 mm) at a 10° angle.

For retrograde monosynaptic tracing, a 1:1 mixture of rAAV‐Ef1α‐DIO‐EGFP‐2A‐TVA‐WPRE‐hGH polyA and rAAV‐CAG‐DIO‐RVG‐WPRE‐hGH polyA (total 150 nL) was unilaterally injected into the CeA of Vgat‐cre mice. After 21‐day viral expression, 100 nL of RV‐CVS‐ENVA‐N2C(^Δ^G)‐tdtomato was injected for circuit mapping and immunofluorescence staining. For anterograde tracing of the PVT‐CeA circuit, rAAV‐CaMKIIa‐EGFP‐WPRE‐hGH polyA (150 nL) was unilaterally injected into the PVT of WT mice, followed by perfusion and brain sectioning (30 µm).

### In Vivo Chemogenetic Manipulations

In hM3Dq‐mediated activation experiments, 40 female C57BL/6J mice were allocated into four groups: mCherry‐NS, mCherry‐CNO, hM3Dq‐NS, and hM3Dq‐CNO, each containing 10 mice. Following at least 21 days of viral expression, CNO (Sigma‐Aldrich, St. Louis, Missouri, USA) was prepared at concentrations of 1 mg mL^−1^ i.p. and 2 mg mL^−1^ for intracranial cannula injections. Behavioral assessments in OFT, EPM, and CPA were preceded by i.p. injections of 1 mg mL^−1^ CNO or NS 15 min prior to testing.

For hM4Di‐mediated inhibition experiments, groups of 10 C57BL/6J mice were divided into DON+mCherry+NS, DON+mCherry+CNO, DON+hM4Di‐NS, and DON+hM4Di+CNO. After 21 days of viral expression, mice underwent oral gavage with 2.5 mg kg^−1^ DON, followed by i.p. injections of 1 mg kg^−1^ CNO or NS before OFT, EPM, and CPA testing. Circuit‐based chemogenetic experiments involved bilateral intracranial cannula injections of 100 nL CNO at 2 mg mL^−1^ (RWD CAT: 62002, 24G needle). Food intake was monitored using standard food pellets by weight during the chemogenetic period.

### In Vivo Optogenetic Procedures

In optogenetic experiments, mice were connected to a light source through bundled optical fibers (RWD, Shenzhen, China). We used the photodetector to monitor and adjust the light intensity throughout the experiments to ensure precise control over the light stimulation. The photodetector allowed real‐time tracking of the light power output, ensuring that the intensity remained consistent during all stimulation periods. The photodetector controlled the stimulation, which enabled us to adjust the light frequency, pulse duration, and intensity. Stimulation protocols involved blue light (470 nm, 15 ms pulse, 20 Hz, 10 mW) with 10‐second light‐on and 10‐second light‐off durations over a 5 min period. For yellow light stimulation (589 nm), continuous illumination at 5–10 mW was employed during the OFT, EPM, and CPA behavioral assessments. Identical stimulation protocols were implemented for control groups, with behavioral evaluations conducted immediately following light stimulation.

### RNA Sequencing

Total RNA was prepared from CeA tissue using Trizol Reagent (B511311, Sangon, China) according to the manufacturer's protocols. The RNA‐seq transcriptome library was prepared following the NovaSeq6000 and High Output v2 kit (Illumina, San Diego, CA, USA) with 1 µg of total RNA. Reference genome and gene model annotation files were downloaded from genome website browser (NCBI/UCSC/Ensembl) directly. Clean reads were mapped to the reference genome by HISAT2 (version 2.0) with default parameters. RSeQC (version 2.6.1) was used to statistics the alignment results. The homogeneity distribution and the genome structure were checked by Qualimap (version 2.2.1). BEDTools (version 2.26.0) was used to statistical analysis the gene coverage ratio. The data generated were analyzed between groups (n = 5 mice per group) for bioinformatics analysis. The Heatmap, Volcano Plot, and Non‐metric Multidimensional Scaling (NMDS) were generated using R 4.3.2 software for differentially expressed genes.

### Quantification and Statistical Analysis

Statistical analyses were executed using GraphPad Prism 8.0, with values presented as mean ± SEM, unless specified differently in figure legends. All independent experiments included a minimum of three biological replicates. Sample sizes were aligned with precedents from similar studies by our group. Two‐group comparisons utilized unpaired Student's t‐tests, reporting P values accordingly. Multigroup analyses employed one‐way or two‐way ANOVA with Bonferroni post‐tests. A threshold of P < 0.05 was established for statistical significance. Detailed statistical data, including exact P values and F values for each figure, are available in Table S1 (Supporting Information).

## Conflict of Interest

The authors declare no conflict of interest.

## Author Contributions

L.N.Y. designed experiments, performed most animal experiments, stereotaxic surgery, electrophysiological recordings, analyzed sequencing data, wrote the paper, and drew the figures. M.M.T. contributed to animal experiments. A.K.N. and L.G.L. supervised the project, W.Y. conceived the study, revised the paper, and supported funding acquisition.

## Supporting information



Supporting Information

Supporting Information

## Data Availability

The data that support the findings of this study are available from the corresponding author upon reasonable request.;

## References

[1] M. W. Schwartz , S. C. Woods , D. Porte Jr. , R. J. Seeley , D. G. Baskin , Nature 2000, 404, 661. 10766253 10.1038/35007534

[2] L. Cifuentes , A. Acosta , Clin Res Hepatol Gastroenterol 2022, 46, 101794. 34481092 10.1016/j.clinre.2021.101794 PMC9721532

[3] Couzin‐Frankel , Science 2020, 368, 124. 32273451 10.1126/science.368.6487.124

[4] J. E. Mitchell , C. B. A. N Peterson , N. Engl. J. Med. 2020, 382, 1343. 32242359 10.1056/NEJMcp1803175

[5] K. A. Knowles , S. C. Jessup , B. O. Olatunji , Curr Psychiatry Rep 2018, 20, 68. 30094516 10.1007/s11920-018-0936-5 PMC6422162

[6] P. Hay , Int J Eat Disord 2013, 46, 462. 23658093 10.1002/eat.22103

[7] M. Strober , Int J Eat Disord 2004, 35, 504. 15101066 10.1002/eat.20029

[8] S. Zipfel , K. E. Giel , C. M. Bulik , P. Hay , U. Schmidt , Lancet Psychiatry 2015, 2, 1099. 26514083 10.1016/S2215-0366(15)00356-9

[9] J. Treasure , A. M. Claudino , N. Zucker , Lancet 2010, 375, 583. 19931176 10.1016/S0140-6736(09)61748-7

[10] G. S. Eriksen , H. K. Knutsen , M. Sandvik , A. L. Brantsæter , Environ Int 2021, 157, 106804. 34352564 10.1016/j.envint.2021.106804

[11] D. Wan , L. Huang , Y. Pan , Q. Wu , D. Chen , Y. Tao , X.u Wang , Z. Liu , J. Li , L. Wang , Z. Yuan , J. Agric. Food Chem. 2014, 62, 288. 24341775 10.1021/jf4047946

[12] J. J. Pestka , E. S. Clark , H. E. Schwartz‐Zimmermann , F. Berthiller , Toxins (Basel) 2017, 9, 240. 31027354 10.3390/toxins11050238 PMC6562573

[13] P. Koletsi , G. F. Wiegertjes , E. A. M. Graat , P. Lyons , J. Schrama , Toxins (Basel) 2022, 14, 810. 36422984 10.3390/toxins14110810 PMC9697072

[14] J. Yue , D. Guo , X. Gao , J. Wang , E. Nepovimova , W. Wu , K. Kuca , Toxins (Basel) 2021, 13, 512. 34437383 10.3390/toxins13080512 PMC8402572

[15] J. J. Pestka , A. T. Smolinski , J Toxicol Environ Health B Crit Rev 2005, 8, 39. 15762554 10.1080/10937400590889458

[16] C. C. Horn , Appetite 2008, 50, 430. 17996982 10.1016/j.appet.2007.09.015 PMC2274963

[17] S. F. Leibowitz , K. E. Wortley , Peptides 2004, 25, 473. 15134868 10.1016/j.peptides.2004.02.006

[18] M. E. Carter , M. E. Soden , L. S. Zweifel , R. D. Palmiter , Nature 2013, 503, 111. 24121436 10.1038/nature12596 PMC3878302

[19] J. A. Hardaway , L. R. Halladay , C. M. Mazzone , D. Pati , D. W. Bloodgood , M. Kim , J. Jensen , J. F. DiBerto , K. M. Boyt , A. Shiddapur , A. Erfani , O. J. Hon , S. Neira , C. M. Stanhope , J. A. Sugam , M. P. Saddoris , G. Tipton , Z. McElligott , T. C. Jhou , G. D. Stuber , M. R. Bruchas , C. M. Bulik , A. Holmes , T. L. Kash , Neuron 2019, 102, 1037. 31029403 10.1016/j.neuron.2019.03.037 PMC6750705

[20] B. Yang , J. Sanches‐Padilla , J. Kondapalli , S. L. Morison , E. Delpire , R. Awatramani , D. J. Surmeier , Neuron 2021, 109, 823. 33476548 10.1016/j.neuron.2020.12.023 PMC9272546

[21] W. Zhou , Y. Jin , Q. Meng , X. Zhu , T. Bai , Y. Tian , Y.u Mao , L. Wang , W. Xie , H. Zhong , N.a Zhang , M.‐H. Luo , W. Tao , H. Wang , J. Li , J. Li , B.‐S. Qiu , J.‐N. Zhou , X. Li , H. Xu , K. Wang , X. Zhang , Y. Liu , G. Richter‐Levin , L. Xu , Z. Zhang , Nat. Neurosci. 2019, 22, 1649. 31451801 10.1038/s41593-019-0468-2

[22] N. Whittle , J. Fadok , K. P. MacPherson , R. Nguyen , P. Botta , S. B. E. Wolff , C. Müller , C. Herry , P. Tovote , A. Holmes , N. Singewald , A. Lüthi , S. Ciocchi , Nat. Commun. 2021, 12, 4156. 34230461 10.1038/s41467-021-24068-x PMC8260764

[23] L. R. Barrett , J. Nunez , X. Zhang , Neuropsychopharmacology 2021, 46, 1045. 33495546 10.1038/s41386-021-00961-3 PMC8114915

[24] G. J. Kirouac , Neurosci Biobehav Rev 2015, 56, 315. 26255593 10.1016/j.neubiorev.2015.08.005

[25] X. Zhang , A. N. van den Pol , Science 2017, 356, 853. 28546212 10.1126/science.aam7100 PMC6602535

[26] J. Meffre , M. Sicre , M. Diarra , F. Marchessaux , D. Paleressompoulle , F. Ambroggi , Curr. Biol. 2019, 29, 3298. 31543448 10.1016/j.cub.2019.07.069

[27] K. Zhou , H. Xu , S. Lu , S. Jiang , G. Hou , X. Deng , M. He , Y. Zhu , Nat. Commun. 2022, 13, 6244. 36271048 10.1038/s41467-022-33843-3 PMC9587247

[28] P. Pliota , V. Böhm , F. Grössl , J. Griessner , O. Valenti , K. Kraitsy , J. Kaczanowska , M. Pasieka , T. Lendl , J. M. Deussing , W. Haubensak , Mol. Psychiatry 2020, 25, 428. 29904149 10.1038/s41380-018-0089-2 PMC6169733

[29] P. H. Janak , K. M. Tye , Nature 2015, 517, 284. 25592533 10.1038/nature14188 PMC4565157

[30] A. L. Mahan , K. J. Ressler , Trends Neurosci. 2012, 35, 24. 21798604 10.1016/j.tins.2011.06.007 PMC3206195

[31] H. Cai , W. Haubensak , T. E. Anthony , D. J. Anderson , Nat. Neurosci. 2014, 17, 1240. 25064852 10.1038/nn.3767 PMC4146747

[32] S. Dodt , N. V. Widdershooven , M.‐L. Dreisow , L. Weiher , L. Steuernagel , F. T. Wunderlich , J. C. Brüning , H. Fenselau , Nat. Commun. 2024, 15, 5439. 38937485 10.1038/s41467-024-49766-0 PMC11211344

[33] É. M. Fekete , K. Inoue , Y.u Zhao , J. E. Rivier , W. W. Vale , A. Szücs , G. F. Koob , E. P. Zorrilla , Neuropsychopharmacology 2007, 32, 1052. 17019404 10.1038/sj.npp.1301214 PMC2748839

[34] C. E. Geisler , L. Décarie‐Spain , M. K. Loh , W. Trumbauer , J. Gaisinsky , M. E. Klug , C. Pelletier , J. F. Davis , H. D. Schmidt , M. F. Roitman , S. E. Kanoski , M. R. Hayes , Biol. Psychiatry 2024, 95, 938. 37517705 10.1016/j.biopsych.2023.07.011 PMC13005266

[35] S. M. Sternson , B. L. Roth , Annu. Rev. Neurosci. 2014, 37, 387. 25002280 10.1146/annurev-neuro-071013-014048

[36] O. B. Levine , M. J. Skelly , J. D. Miller , J. K. Rivera‐Irizarry , S. A. Rowson , J. F. DiBerto , J. A. Rinker , T. E. Thiele , T. L. Kash , K. E. Pleil , Nat. Commun. 2021, 12, 5080. 34426574 10.1038/s41467-021-25368-y PMC8382748

[37] S.‐H. Liang , W.‐J. Zhao , J.‐B. Yin , Y.‐B. Chen , J.‐N. Li , B. Feng , Y.‐C. Lu , J. Wang , Y.‐L. Dong , Y.‐Q. Li , J. Neurosci. 2020, 40, 7837. 32958568 10.1523/JNEUROSCI.2487-19.2020 PMC7548696

[38] S. Ren , Y. Wang , F. Yue , X. Cheng , R. Dang , Q. Qiao , X. Sun , X. Li , Q. Jiang , J. Yao , H. Qin , G. Wang , X. Liao , D. Gao , J. Xia , J. Zhang , B.o Hu , J. Yan , Y. Wang , M. Xu , Y. Han , X. Tang , X. Chen , C. He , Z. Hu , Science 2018, 362, 429. 30361367 10.1126/science.aat2512

[39] K. Li , T. Zhou , L. Liao , Z. Yang , C. Wong , F. Henn , R. Malinow , J. R. Yates , H. Hu , Science 2013, 341, 1016. 23990563 10.1126/science.1240729 PMC3932364

[40] R. A. Nicoll , H. Schulman , Physiol. Rev. 2023, 103, 2897. 10.1152/physrev.00034.2022 PMC10642921 37290118

[41] J. E. Tullis , M. E. Larsen , N. L. Rumian , R. K. Freund , E. E. Boxer , C. N. Brown , S. J. Coultrap , H. Schulman , J. Aoto , M. L. Dell'Acqua , K. U. Bayer , Nature 2023, 621, 146. 37648853 10.1038/s41586-023-06465-y PMC10482691

[42] K. R. Taylor , T. Barron , A. Hui , A. Spitzer , B. Yalçin , A. E. Ivec , A. C. Geraghty , G. G. Hartmann , M. Arzt , S. M. Gillespie , Y. S. Kim , S. Maleki Jahan , H. Zhang , K. Shamardani , M. Su , L. Ni , P. P. Du , P. J. Woo , A. Silva‐Torres , H. S. Venkatesh , R. Mancusi , A. Ponnuswami , S. Mulinyawe , M. B. Keough , I. Chau , R. Aziz‐Bose , I. Tirosh , M. L. Suvà , M. Monje , Nature 2023, 623, 366. 37914930 10.1038/s41586-023-06678-1 PMC10632140

[43] M. A. Penzo , C. Gao , Trends Neurosci. 2021, 44, 538. 33775435 10.1016/j.tins.2021.03.001 PMC8222078

[44] K. Zhou , Y. Zhu , Pharmacol Res 2019, 142, 70. 30772461 10.1016/j.phrs.2019.02.014

[45] W. Wu , H.‐R. Zhou , K. He , X. Pan , Y. Sugita‐Konishi , M. Watanabe , H. Zhang , J. J. Pestka , Toxicol. Sci. 2014, 138, 278. 24385417 10.1093/toxsci/kft335 PMC4246666

[46] F. Wu , J. D. Groopman , J. J. Pestka , Annu. Rev. Food Sci. Technol. 2014, 5, 351. 24422587 10.1146/annurev-food-030713-092431

[47] W. Wu , B. M. Flannery , Y. Sugita‐Konishi , M. Watanabe , H. Zhang , J. J. Pestka , Food Chem. Toxicol. 2012, 50, 2056. 22465835 10.1016/j.fct.2012.03.055

[48] B. M. Flannery , E. S. Clark , J. J. Pestka , Toxicol. Sci. 2012, 130, 289. 22903826 10.1093/toxsci/kfs255 PMC3621367

[49] E. S. Clark , B. M. Flannery , J. J. M. A. R. D Pestka , Toxins (Basel) 2015, 7, 2845. 26230710 10.3390/toxins7082845 PMC4549728

[50] B. M. Flannery , W. Wu , J. J. Pestka , Food Chem. Toxicol. 2011, 49, 1863. 21575669 10.1016/j.fct.2011.05.004 PMC3124119

[51] E. S. Clark , B. M. Flannery , E. M. Gardner , J. J. Pestka , Toxins (Basel) 2015, 7, 4199. 26492270 10.3390/toxins7104199 PMC4626729

[52] Y. Fan , R. K. Støving , S. Berreira Ibraim , T. Hyötyläinen , F. Thirion , T. Arora , L. Lyu , E. Stankevic , T. H. Hansen , P. Déchelotte , T. Sinioja , O. Ragnarsdottir , N. Pons , N. Galleron , B. Quinquis , F. Levenez , H. Roume , G. Falony , S. Vieira‐Silva , J. Raes , L. Clausen , G. K. Telléus , F. Bäckhed , M. Oresic , S. D. Ehrlich , O. Pedersen , Nat. Microbiol. 2023, 8, 787. 37069399 10.1038/s41564-023-01355-5 PMC10159860

[53] M. Schroeder , M. Jakovcevski , T. Polacheck , Y. Drori , A. Luoni , S. Röh , J. Zaugg , S. Ben‐Dor , C. Albrecht , A. Chen , Nat. Commun. 2018, 9, 1596. 29686286 10.1038/s41467-018-03836-2 PMC5913294

[54] P. C. Keyes , E. L. Adams , Z. Chen , L. Bi , G. Nachtrab , V. J. Wang , M. Tessier‐Lavigne , Y. Zhu , X. Chen , Neuron 2020, 107, 1113. 32679036 10.1016/j.neuron.2020.06.028 PMC8130576

[55] J. Ma , J. du Hoffmann , M. Kindel , B. S. Beas , Y. Chudasama , M. A. Penzo , Nat. Neurosci. 2021, 24, 1429. 34413514 10.1038/s41593-021-00912-7 PMC8484052

[56] G. P. McNally , Neurosci Biobehav Rev 2021, 125, 193. 33609570 10.1016/j.neubiorev.2021.02.021

[57] H. Li , P. Namburi , J. M. Olson , M. Borio , M. E. Lemieux , A. Beyeler , G. G. Calhoon , N. Hitora‐Imamura , A. A. Coley , A. Libster , A. Bal , X. Jin , H. Wang , C. Jia , S. R. Choudhury , X.i Shi , A. C. Felix‐Ortiz , V. de la Fuente , V. P. Barth , H. O. King , E. M. Izadmehr , J. S. Revanna , K. Batra , K. B. Fischer , L. R. Keyes , N. Padilla‐Coreano , C. A. Siciliano , K. M. McCullough , R. Wichmann , K. J. Ressler , et al., Nature 2022, 608, 586. 35859170 10.1038/s41586-022-04964-y PMC9583860

[58] C. Gao , Y. Leng , J. Ma , V. Rooke , S. Rodriguez‐Gonzalez , C. Ramakrishnan , K. Deisseroth , M. A. Penzo , Nat. Neurosci. 2020, 23, 217. 31932767 10.1038/s41593-019-0572-3 PMC7007348

[59] F. Yuan , Z. Zhou , S. Wu , F. Jiao , L. Chen , L. Fang , H. Yin , X. Hu , X. Jiang , K. Liu , F. Xiao , H. Jiang , S. Chen , Z. Liu , Y. Shu , F. Guo , Proc Natl Acad Sci U S A 2023, 120, e2215590120. 37126693 10.1073/pnas.2215590120 PMC10175747

[60] T. Hua , B. Chen , D. Lu , K. Sakurai , S. Zhao , B.‐X. Han , J. Kim , L. Yin , Y. Chen , J. Lu , F. Wang , Nat. Neurosci. 2020, 23, 854. 32424286 10.1038/s41593-020-0632-8 PMC7329612

[61] T. Yang , K. Yu , X. Zhang , X. Xiao , X. Chen , Y.u Fu , B. Li , Nature 2023, 616, 510. 37020025 10.1038/s41586-023-05910-2 PMC10665639

[62] A. L. Alhadeff , R. A. Holland , H. Zheng , L. Rinaman , H. J. Grill , B. C. De Jonghe , J. Neurosci. 2017, 37, 362. 28077715 10.1523/JNEUROSCI.2714-16.2016 PMC5242394

[63] A. Iemolo , A. Ferragud , P. Cottone , V. Sabino , Neuropsychopharmacology 2015, 40, 1846. 25649277 10.1038/npp.2015.34 PMC4839508

[64] M. R. Sanchez , Y. Wang , T. S. Cho , W. I. Schnapp , M. B. Schmit , C. Fang , H. Cai , Mol. Metab. 2022, 58, 101443. 35066159 10.1016/j.molmet.2022.101443 PMC8844644

[65] H. Jiang , J.‐P. Liu , K.e Xi , L.‐Y.u Liu , L.‐Y.u Kong , J. Cai , S.i‐Q. Cai , X.i‐Y. Han , J.‐G. Song , X.‐M. Yang , Y. Wan , G.‐G. Xing , J. Neurosci. 2021, 41, 7278. 34272314 10.1523/JNEUROSCI.2678-20.2021 PMC8387122

[66] J. Tzschoppe , F. Nees , T. Banaschewski , G. J. Barker , C. Büchel , P. J. Conrod , H. Garavan , A. Heinz , E. Loth , K. Mann , J.‐L. Martinot , M. N. Smolka , J. Gallinat , A. Ströhle , M. Struve , M. Rietschel , G. Schumann , H. Flor , Neuropsychopharmacology 2014, 39, 875. 24126454 10.1038/npp.2013.287 PMC3924522

[67] J. Griessner , M. Pasieka , V. Böhm , F. Grössl , J. Kaczanowska , P. Pliota , D. Kargl , B. Werner , N. Kaouane , S. Strobelt , S. Kreitz , A. Hess , W. Haubensak , Mol. Psychiatry 2021, 26, 534. 30504824 10.1038/s41380-018-0310-3 PMC6411154

[68] Z. Zhou , X. Liu , S. Chen , Z. Zhang , Y. Liu , Q. Montardy , Y. Tang , P. Wei , N. Liu , L. Li , R.u Song , J. Lai , X. He , C. Chen , G. Bi , G. Feng , F. Xu , L. Wang , Neuron 2019, 103, 473. 31202540 10.1016/j.neuron.2019.05.027

[69] Q. Ye , J. Nunez , X. Zhang , Sci. Adv. 2023, 9, eadi5326. 37976360 10.1126/sciadv.adi5326 PMC10656063

[70] Y. B. Zhu , Y. Wang , X. X. Hua , L. Xu , M. Z. Liu , R. Zhang , P. F. Liu , J. B. Li , L. Zhang , D. Mu , Elife 2022, 11, e68372. 35167440 10.7554/eLife.68372 PMC8929929

[71] D. Liu , J. Li , J. Wu , J. Dai , X. Chen , Y. Huang , S. Zhang , B.o Tian , W. Mei , Front Neural Circuits 2020, 14, 55. 32973462 10.3389/fncir.2020.00055 PMC7461971

[72] J. J. Chemali , C. J. Van Dort , E. N. Brown , K. Solt , Anesthesiology 2012, 116, 998. 22446983 10.1097/ALN.0b013e3182518bfc PMC3339625

[73] S. Kantor , L. Szabo , J. Varga , M. Cuesta , A. J. Morton , Brain 2013, 136, 2147. 23801737 10.1093/brain/awt128

[74] C. Jiang , X. Yang , G. He , F. Wang , Z. Wang , W. Xu , Y. Mao , L. Ma , F. Wang , Mol. Psychiatry 2021, 26, 6170. 34642456 10.1038/s41380-021-01321-9 PMC8760059

[75] H. Jia , N. L. Rochefort , X. Chen , A. Konnerth , Nat. Protoc. 2011, 6, 28. 21212780 10.1038/nprot.2010.169

[76] C. K. Kim , S. J. Yang , N. Pichamoorthy , N. P. Young , I. Kauvar , J. H. Jennings , T. N. Lerner , A. Berndt , S. Y. Lee , C. Ramakrishnan , T. J. Davidson , M. Inoue , H. Bito , K. Deisseroth , Nat. Methods 2016, 13, 325. 26878381 10.1038/nmeth.3770 PMC5717315

