# Supplementary material for: Regulation of PVT‐CeA Circuit in Deoxynivalenol‐Induced Anorexia and Aversive‐Like Emotions
Source: Adv Sci (Weinh). 2025 Feb 28;12(16):2417068. doi: 10.1002/advs.202417068 (PMC12021098; doi:10.1002/advs.202417068)
Supplement: Supplementary file 1 — Supporting Information [file ADVS-12-2417068-s002.docx]

**Supplementary Information**

**PVT-CeA Circuit Regulation of Deoxynivalenol-Induced Anorexia and Aversive-like Emotions**

Liu-Nan Yang^1,2,3^ Mingmeng Tang^1,2,3^ Andreas K. Nüssler^4^ Liegang Liu^1,2,3^ Wei Yang^1,2,3*^

*: Corresponding author.

This file includes:

Supplementary material and methods;

Supplementary figures and legends: Figure S1 to S9

**Supplementary** **Materials and Methods**

**In Vivo Multi-Channel Recording**

We employed an 800-channel, custom-built, non-removable multi-channel electrode system to record local field potential (LFP) discharge frequencies in the CeA region. These LFP signals were amplified with a gain of 1,000 and underwent simulated band-pass filtering between 1.5-1700 Hz using a Type 0 differential AC amplifier (A-M Systems, Sequim, WA, USA). The filtered signals (1-80 Hz) at a sampling rate of 1,000 Hz were digitized and stored for subsequent offline analysis. LFP alterations were evaluated for 60 seconds before and after light administration. We calculated the average power spectral density (PSD) across the entire frequency range (1-50 Hz) utilizing the Welch method. Additionally, to mitigate external power supply interference, we excluded the 48-52 Hz power range from our analysis.

**Paired-Pulse Stimulation Recordings**

Paired-pulse stimulation recordings were conducted in two groups: the Vehicle (saline) group and the DON-treated group. A bipolar stimulating electrode was placed in the CeA, and paired stimuli with a duration of 0.2 ms were delivered at an inter-stimulus interval (ISI) of 50 ms using a constant-current stimulus isolation unit. The stimulation intensity was adjusted to evoke an EPSC amplitude equal to 30% of the maximum response. Evoke EPSC was recorded using glass micropipettes filled with intrapipette solution (1–2 MΩ), The intrapipette solution used throughout the entire study contained (in mM): 100 Cs-methanesulfonate,10 CsCl, 5NaCl, 1MgCl2, 5Tetraethylammonium-Cl, 8EGTA, 1CaCl2, 10HEPES, 5QX-314, 3NaATP, 0.2MgGTP, and spermine tetrahydrochloride (0.1 mM). amplified, digitized at 10 kHz, and analyzed using appropriate software. The Paired-Pulse Ratio (PPR) was calculated as the ratio of the amplitude of the second EPSC to that of the first EPSC.

PPR=Amplitude of FP1​/Amplitude of FP2​​

Data were expressed as means ± SEM and statistical comparisons between the two group were performed using Student’s t-test to determine any significant differences.

**Supplementary Figure and Figure legends**

**
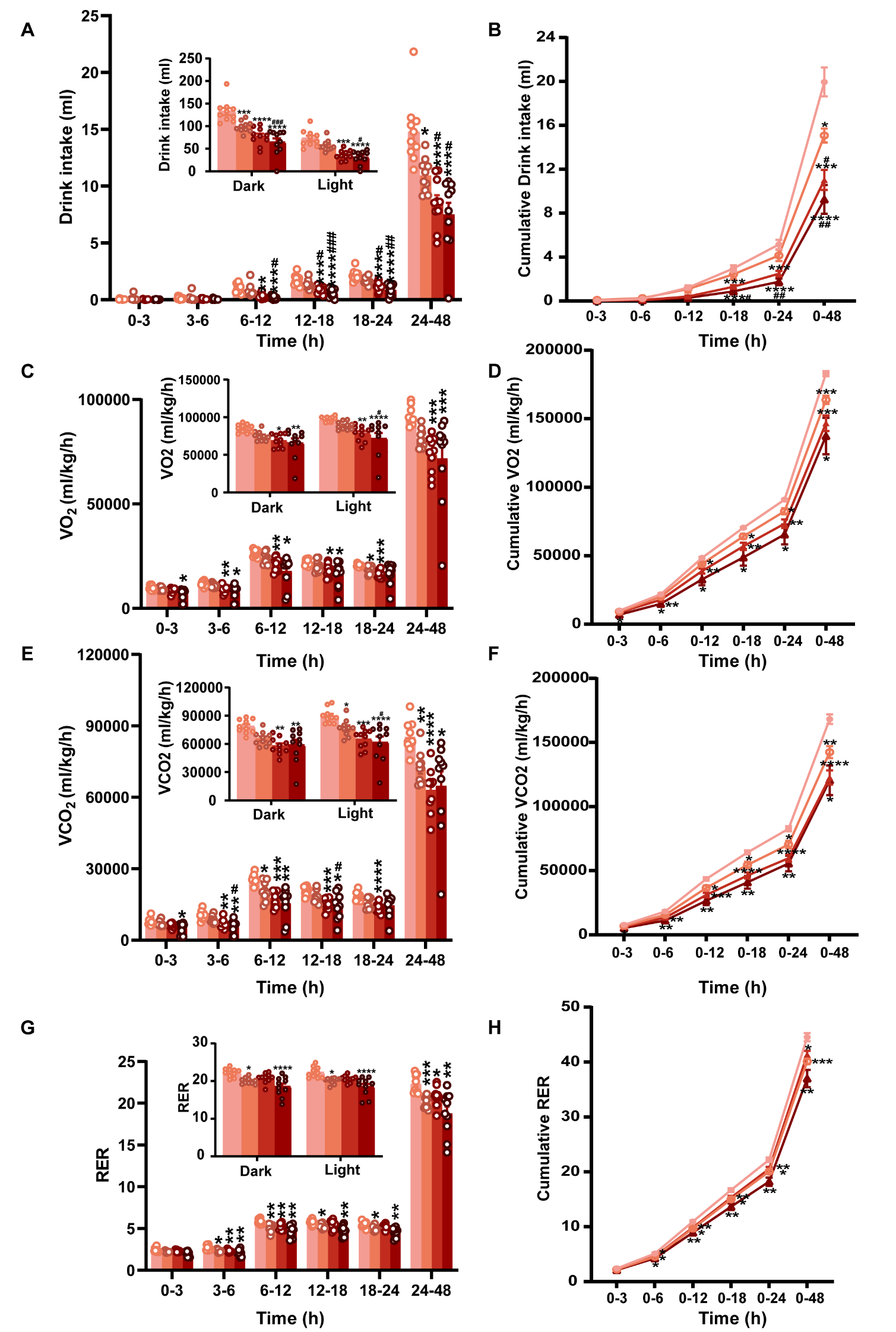
**

**Figure S1 Other DON-Induced Physiological** **indicators**

(A-B) Drink Intake: Tracked at intervals (A) and cumulatively (B) over 48 hours in mice treated with vehicle or DON (1, 2.5 and 5 mg/kg) orally. Inset details nocturnal/diurnal food intake. (C-D) VO_2_ consumption: Assessed at intervals (C) and cumulatively (D) over 48 hours. Inset shows nocturnal/diurnal VO_2_. (E-F) VCO_2_ consumption: Assessed at intervals (E) and cumulatively (F) over 48 hours. Inset shows nocturnal/diurnal VCO_2_. (G-H) RER: Assessed at intervals (G) and cumulatively (H) over 48 hours. Inset shows nocturnal/diurnal RER. n = 10 mice/group. Statistical Methods: Employed two-way ANOVA for data analysis.


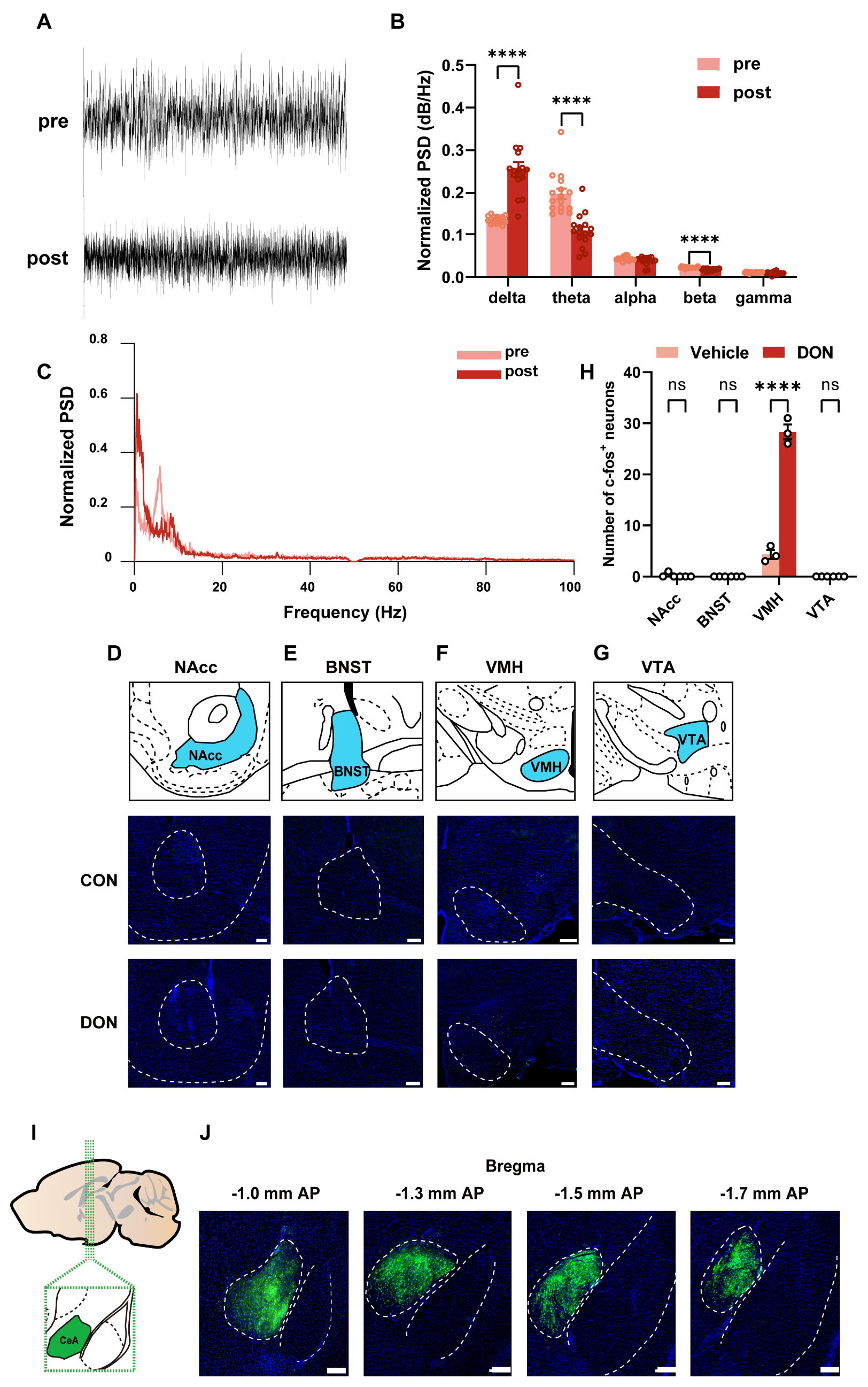


**Figure S2 Effects of DON in** **Local Field Potentials Recordings in CeA**

(A) Representative traces of electrophysiology pre and post DON. (B) Normalized PSD quantification. (C) PSD Analysis: Power spectral density in Vehicle and DON-treated groups (2.5 mg/kg). Statistical analysis using two-way ANOVA (*p* values: interaction *p* <0.0001, time *p* =0.2487, group *p* =<0.0001. n = 16 mice/group). Fluorescence expression of DON in the nucleus accumbens (NAcc) (D), bed nucleus of the stria terminalis (BNST) (E), ventromedial hypothalamus (VMH) (F), and ventral tegmental area (VTA) (G) brain regions. Scale bar = 100 μm. (H) The statistical analysis of c-fos-positive cells in various brain regions of the DON group (Welch-corrected two-tailed unpaired t-test) shows the following results compared to the control group: NAcc (*p* = 0.9925), BNST (*p* > 0.9999), VMH (*p* < 0.0001), VTA (*p* > 0.9999). n = 3 mice per group. (I) Illustration of anatomical localization of four coronal sections of target CeA. (J) Representative fluorescent images of CeA sections in (I). Scale bar = 200 μm.


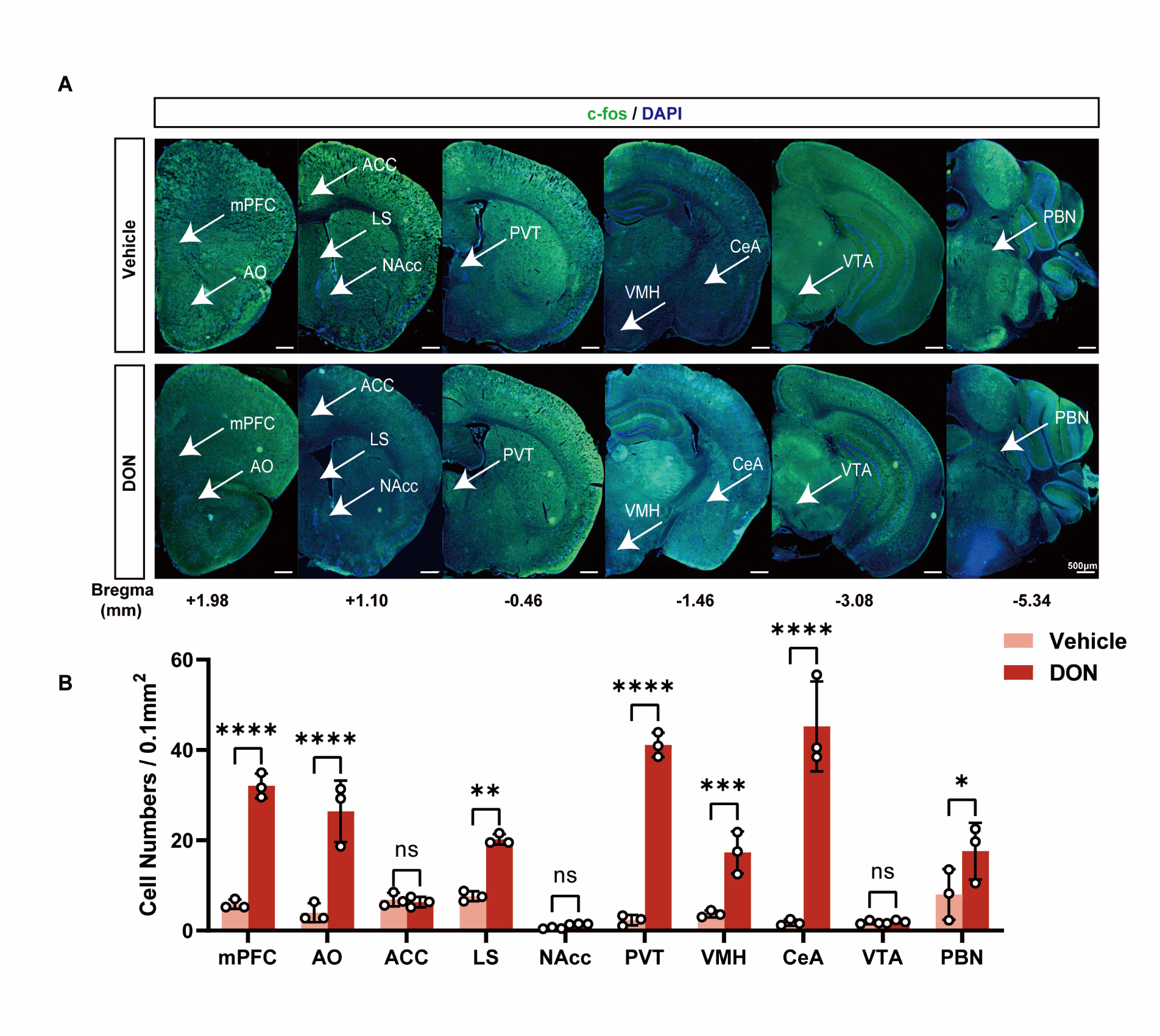


**Figure S3 Whole-brain c-fos Expression**

(A) Representative images of whole-brain c-fos expression in the Vehicle group (Saline) and the DON group (2.5 mg/kg DON). (B) Quantification of c-fos expression is shown in (A). Statistical analysis was performed using a two-tailed unpaired t-test (n = 3). Data are presented as mean ± SEM. **p* < 0.05, ***p* < 0.01, ****p* < 0.001. Scale bar = 500 μm.


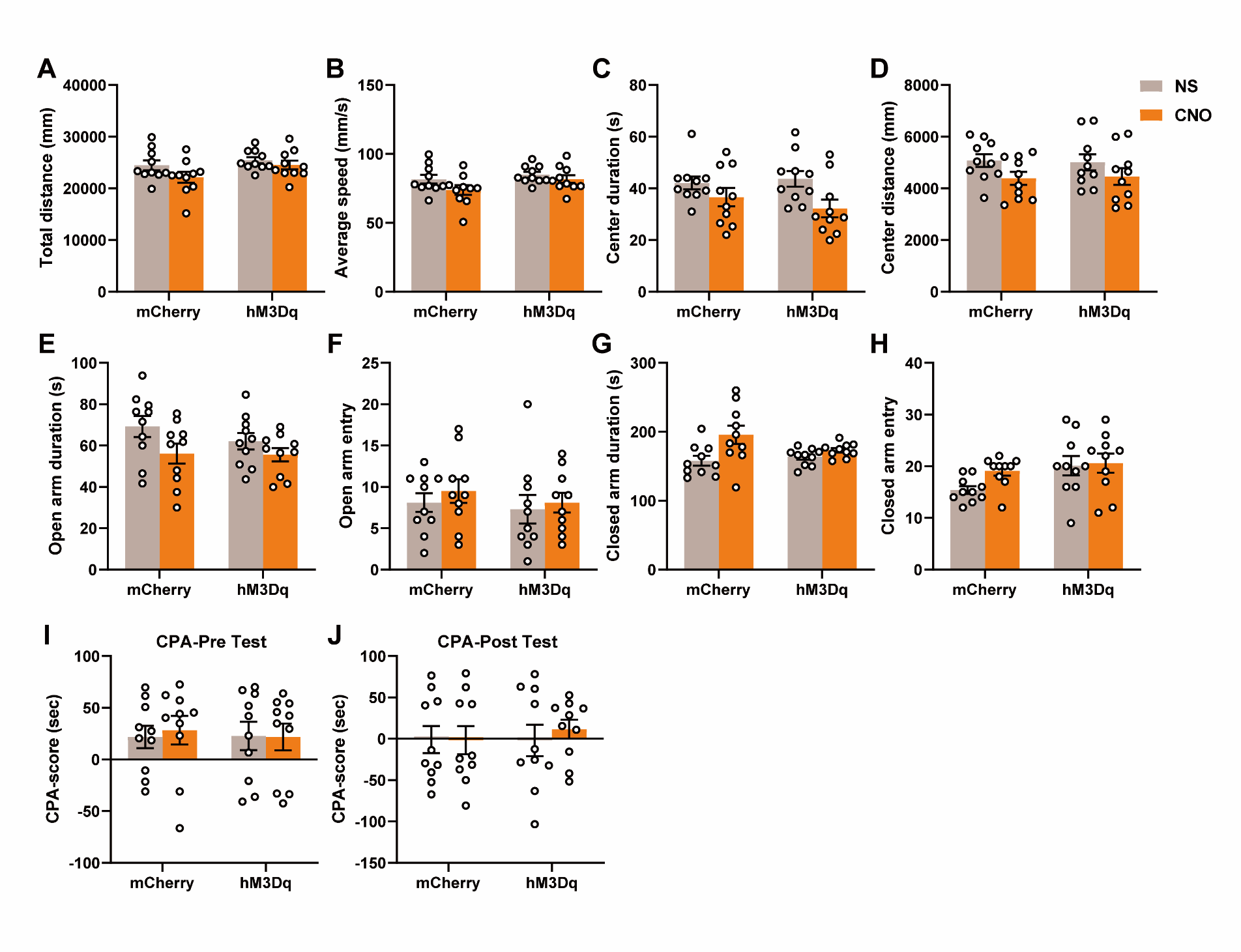


**Figure S4 Activation of VMH Neurons Does Not Induce Anxiety or Aversive-like Behavior in Mice**

AAV2/9-hSyn-hM3Dq-mCherry and AAV9-DIO-mCherry viruses were injected into the VMH nucleus of WT mice to investigate behavioral effects. Behavioral parameters were assessed through multiple tests: total distance traveled (A) and average speed (B) in the OFT, as well as time spent in the center zone (C) and distance traveled in the center zone (D) of the OFT. In the EPM, open arm entries (E), time spent in closed arms (F, G), and the number of closed arm entries (H) were measured. Additionally, pre-test (I) and post-test (J) results in the CPA test were analyzed. Statistical analysis was performed using one-way ANOVA (n = 10 mice per group).

**
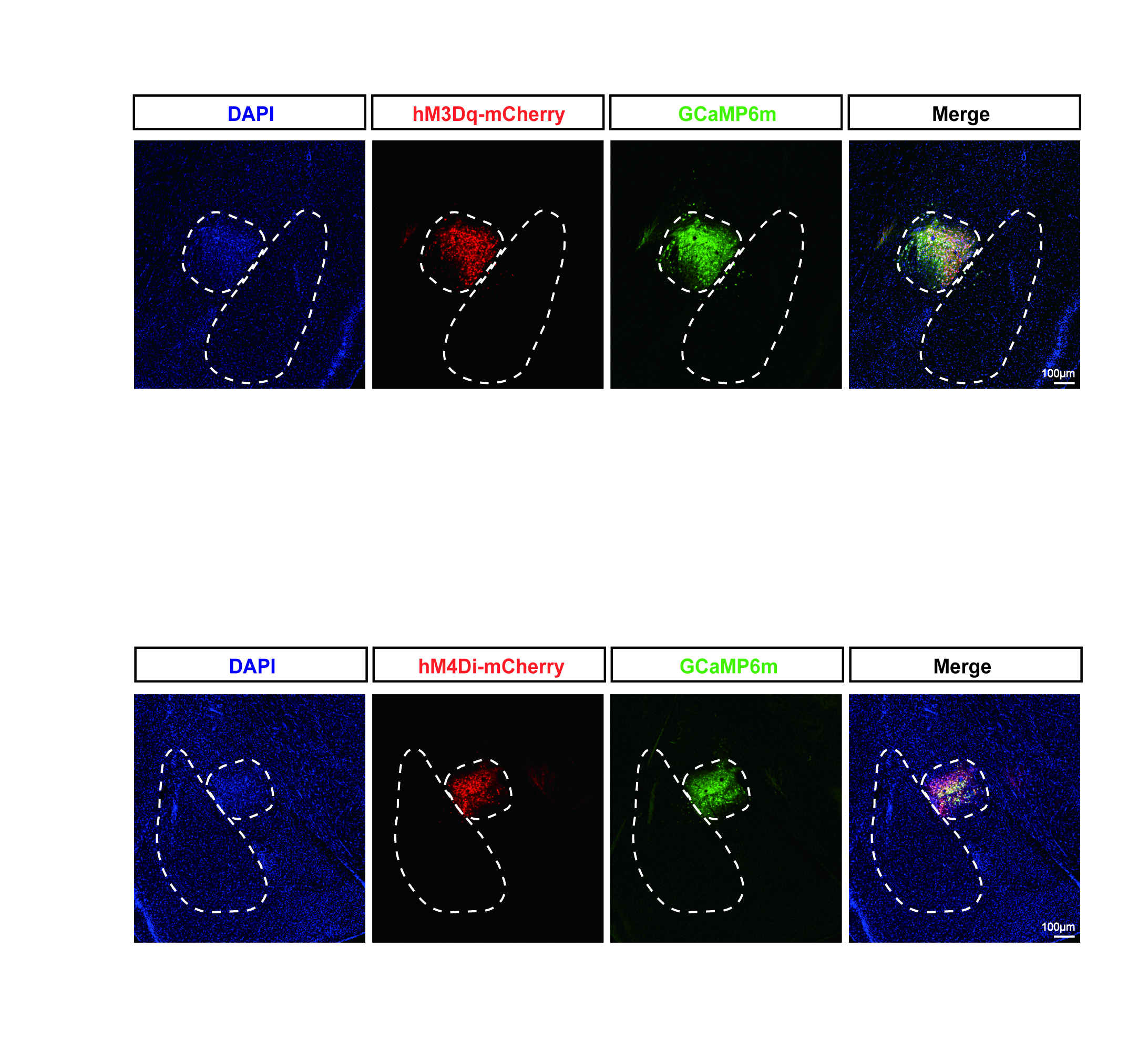
**

**Figure S5 Chemogenetic Activation of CeA GABAergic Neurons and Calcium Imaging Validation**In Vgat-Cre mice, the CeA region was co-infected with AAV9-DIO-hM3Dq-mCherry and AAV2/9-DIO-GCaMP6m viruses to achieve chemogenetic activation. Calcium imaging confirmed co-localization of hM3Dq (Red) and GCaMP6m (Green). Scale bar = 100 μm.

**
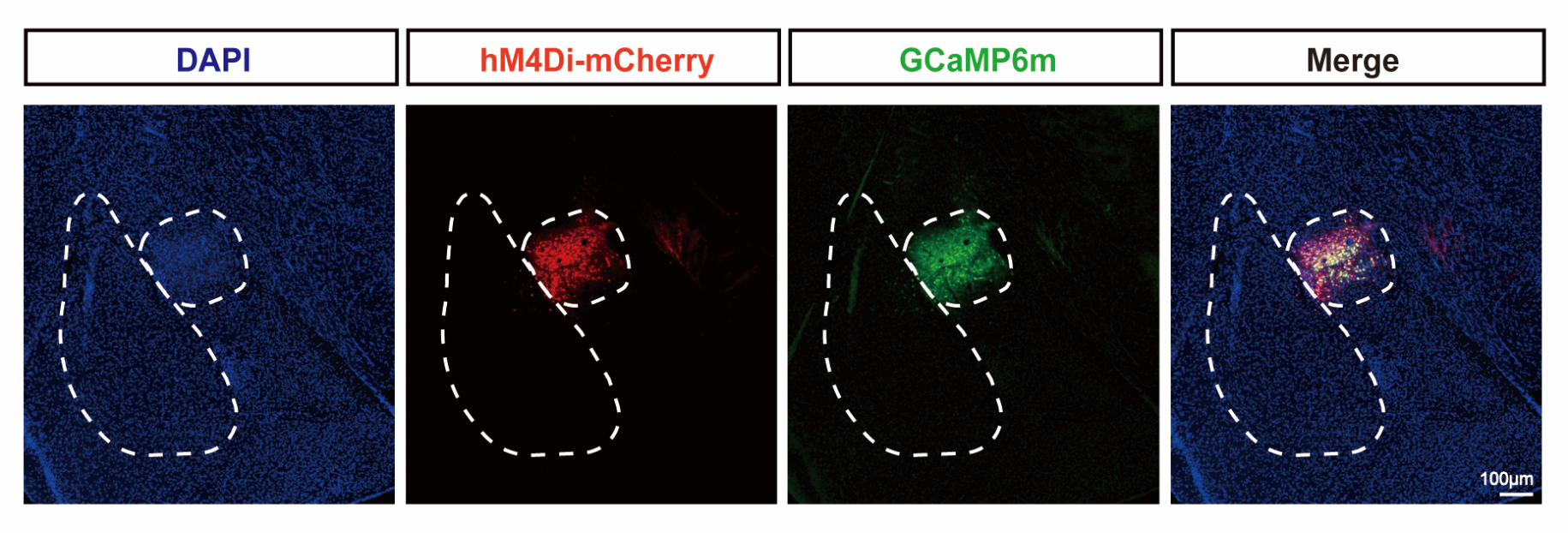
**

**Figure S6 Chemogenetic Inhibition of CeA GABAergic Neurons and Calcium Imaging Validation**In Vgat-Cre mice, the CeA region was co-infected with AAV9-DIO-hM4Di-mCherry and AAV2/9-DIO-GCaMP6m viruses to achieve chemogenetic inhibition. Calcium imaging confirmed co-localization of hM4Di (Red) and GCaMP6m (Green). Scale bar = 100 μm.

**
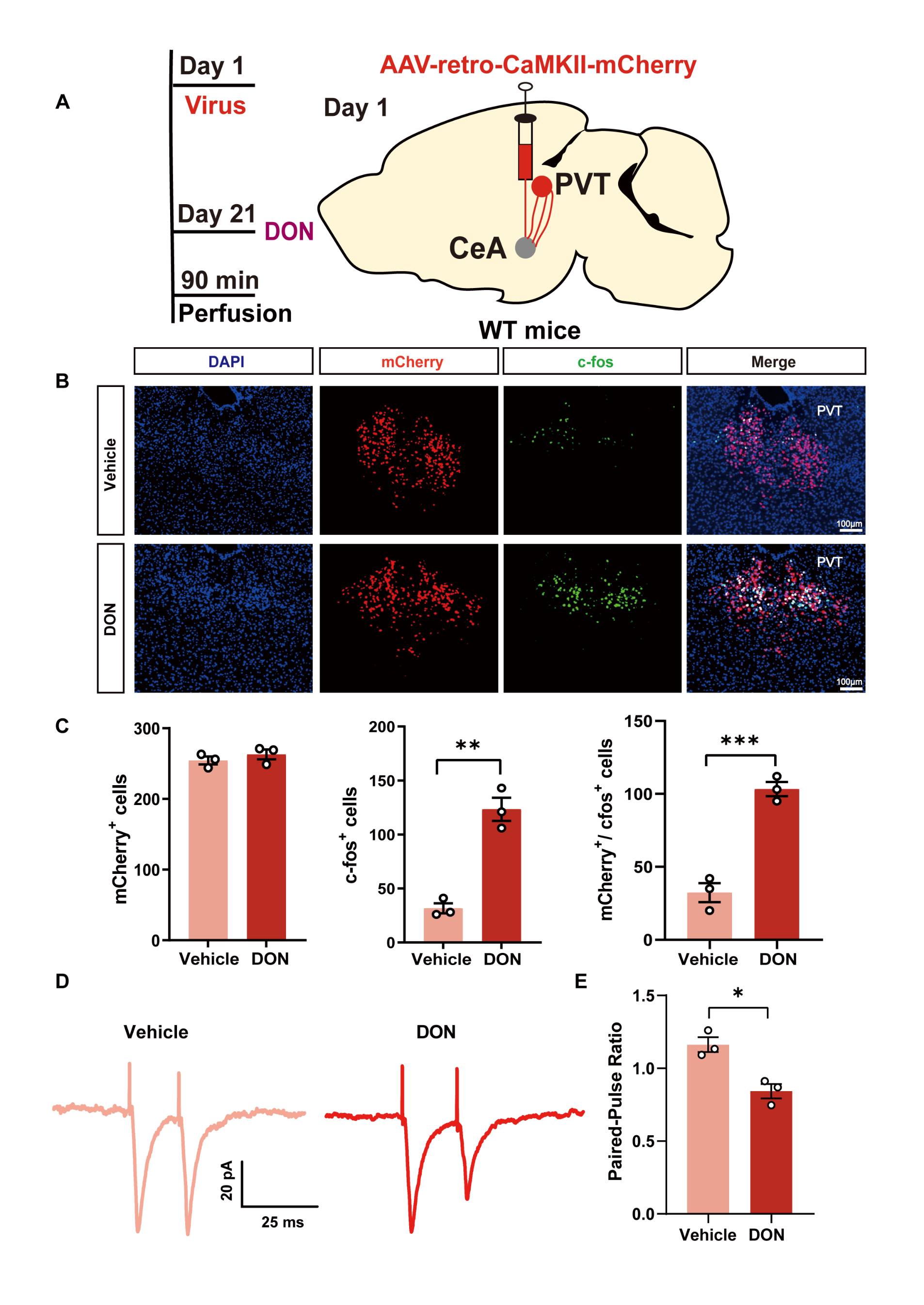
**

**Figure S7 CeA-to-PVT Projections and Functional Connectivity Under DON Exposure**

(A) Schematic Diagram of Viral Injection. (B) Retrograde labeling with AAV-retro-mCherry injected into the CeA demonstrates projections from the CeA to the PVT under DON exposure. (C) c-Fos staining in the PVT reveals overlap with CeA projections under DON conditions. (D) Paired-pulse ratio (PPR) experiments indicate significantly increased functional connectivity between the PVT and CeA following DON exposure. (E) Quantification of (D). Statistical analysis was performed using a two-tailed unpaired t-test (n = 3). Data are presented as mean ± SEM. **p* < 0.05, ***p* < 0.01, ****p* < 0.001. Scale bar = 100 μm.

_
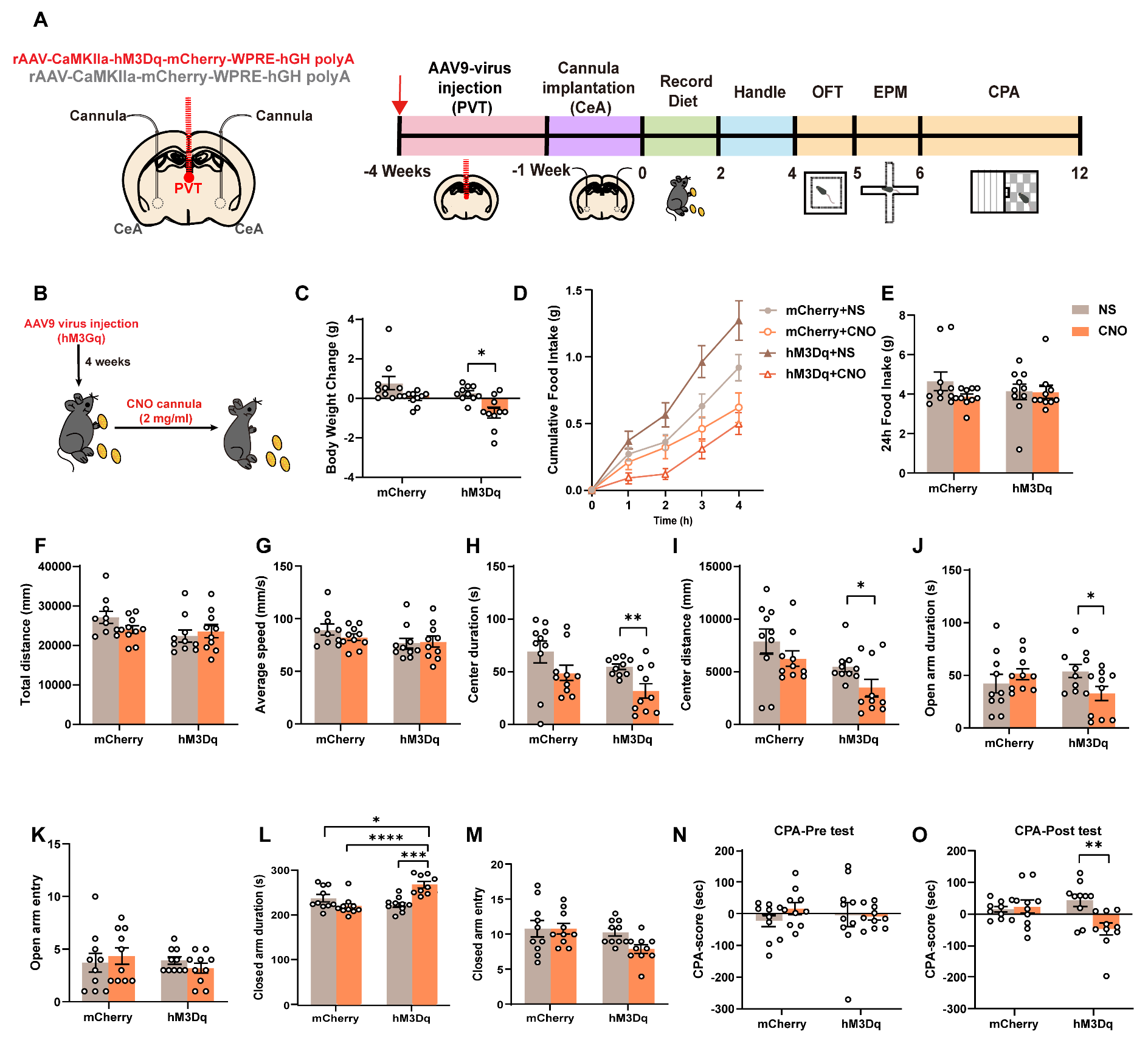
_

**Figure S8 Chemogenetic Activation of PVT-CeA Triggers Anorexia and Aversive-like Emotions**

(A) Viral Injection: AAV9-hM3Dq-mCherry and AAV9-DIO-mCherry viruses Injected into PVT nucleus, followed by cannula embedding in CeA after 3 weeks in WT mice. Accompanied by experimental schemes and timelines (right). (B) Food Intake Schematic: Post-CNO administration. (C) Body Weight Changes in 24 hours. (D) Cumulative Food Intake in 4 hours. (E) Food Intake at 24 hours in four groups. (F) Total Distance in OPT. (G) Average Speed in OPT. (H) Center Duration in OPT. (I) Center Distance in OPT. (J) Open Arm Entry in EPM. (K) Close Arm Duration in EPM. (L) Close Arm Duration in EPM. (M) Close Arm Entry in EPM. (N) Pre-Test in CPA. (O) Post-Test in CPA. n = 10 mice/group. Statistical Methods: Employed one-way ANOVA (C, E-O), and two-way ANOVA (D) for data analysis.


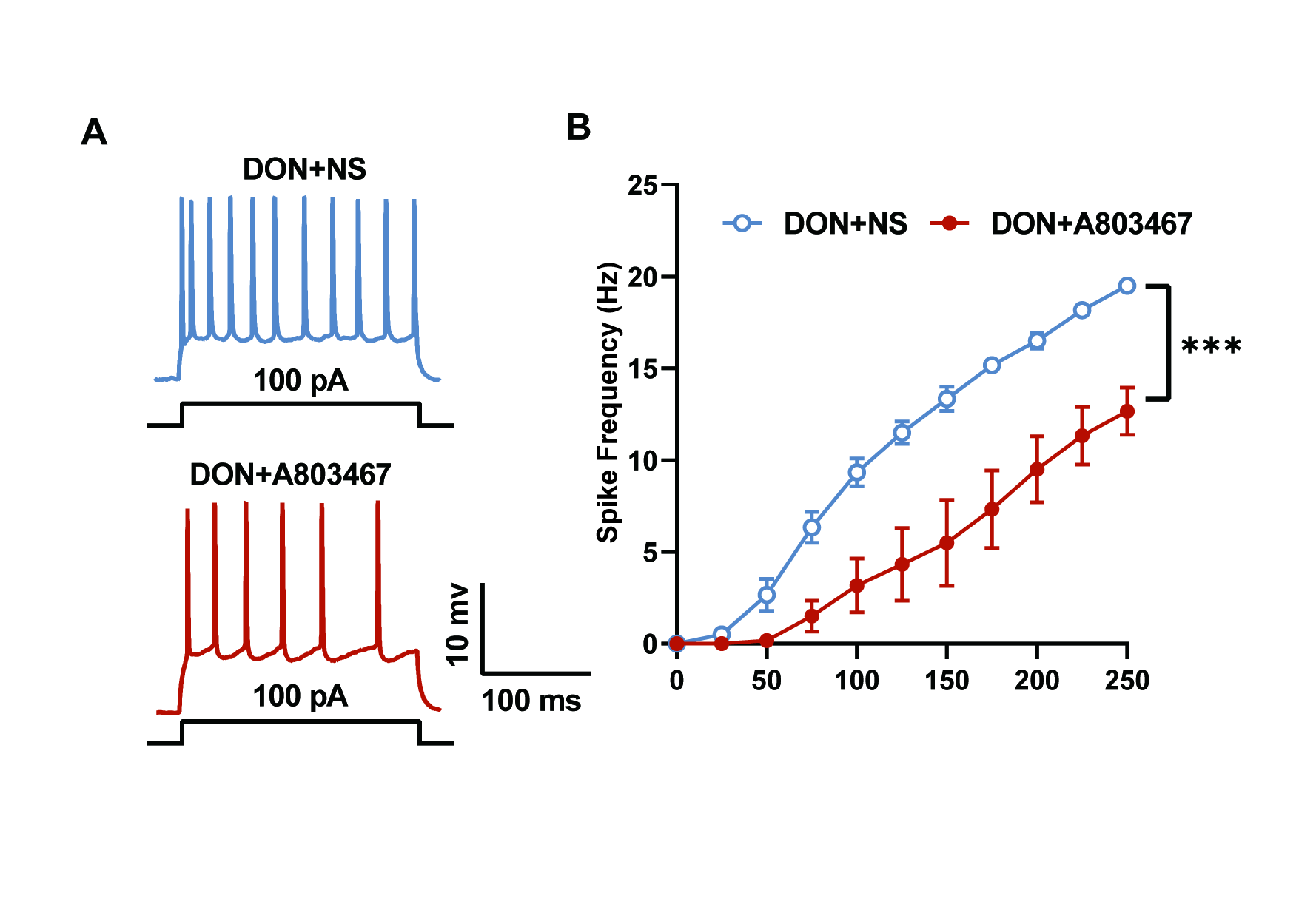


**Figure S9 Effect of SCN10A Inhibition on DON-induced CeA Neuronal Activity
(A)** Representative patch-clamp recordings of action potentials in CeA neurons under DON+NS (blue) and DON+A803467 (red) conditions. **(B)** Quantification of action potential frequency under the two conditions. Statistical analysis was performed using two-way ANOVA (n = 6). Data are presented as mean ± SEM. ****p* < 0.001. Scale bar = 100 μm.
